# Intervention characteristics and speech therapy strategies in care for autistic children in health services: a scoping review

**DOI:** 10.1590/2317-1782/e20250012en

**Published:** 2025-12-08

**Authors:** Renata Chrystina Bianchi de Barros, Eric Batista Ferreira, Priscila Mara Ventura Amorim Silva, Mariana Minante Khalil, Ana Paula de Moraes Oliveira

**Affiliations:** 1 Departamento de Desenvolvimento Humano e Reabilitação, Faculdade de Ciências Médicas, Universidade Estadual de Campinas – UNICAMP - Campinas (SP), Brasil.; 2 Departamento de Estatística, Universidade Federal de Alfenas – UNIFAL-MG – Alfenas (MG), Brasil.; 3 Centro de Estudos e Pesquisas em Reabilitação “Prof. Dr. Gabriel O.S. Porto” – CEPRE, Universidade Estadual de Campinas – UNICAMP - Campinas (SP), Brasil.; 4 Faculdade de Medicina, Universidade Federal de Alfenas – UNIFAL-MG - Alfenas (MG), Brasil.; 5 Biblioteca, Universidade Estadual de Campinas – UNICAMP - Campinas (SP), Brasil.

**Keywords:** Autism, Autism Spectrum Disorder, Rehabilitation of Speech and Language Disorders, Speech-Language Pathology, Brazil

## Abstract

**Purpose:**

to identify and systematize speech therapy practices with autistic children in healthcare services reported as an evaluative and therapeutic strategy in Brazil.

**Research strategies:**

a scoping review following the guidelines of the Joanna Briggs Institute and PRISMA-ScR. Articles, essays, reviews, and gray literature available until July 4, 2024, were retrieved from databases such as PubMed, Scielo, Scopus, Web of Science, ProQuest Central, Embase, EBSCOhost, BVS, BDTD, and Google Scholar. Reference lists and relevant systematic reviews were also checked for additional documents.

**Selection criteria:**

based on the PCC format (Participants: autistic children aged 2 to 12 years; Concept: speech therapy assessment and treatment strategies; Context: Brazil).

**Data analysis:**

data were extracted using a pre-designed matrix considering author, type/year of publication, objective, sample, autism concept, type/strategy of intervention, setting, and conclusion. Descriptive quantitative and qualitative analyses were performed.

**Results:**

a total of 49 studies were included in the review, allowing the identification that speech therapy practices targeting autistic children in Brazilian healthcare services predominantly involve therapeutic and evaluative approaches, mainly carried out in university clinics.

**Conclusion:**

although speech therapy has advanced in the personalization of care and adaptation of therapeutic strategies, there is still a predominance of interventions focused on diagnosis and rehabilitation, with a limited number of studies addressing health promotion and social inclusion actions.

## INTRODUCTION

The role and importance of the speech-language pathologist in the care of individuals with Autism Spectrum Disorder (ASD) is recognized as a central element in the development of communication and social skills, which are frequently impaired in individuals with this condition^(1)^. Autism Spectrum Disorder is a developmental disorder characterized^(2,3)^ by severe and persistent deficits in social communication and interaction, in addition to restricted and repetitive behaviors, requiring specialized and individualized interventions to address the specific needs of each individual. In this context, health services play a crucial role in organizing and providing assessment and therapeutic strategies that ensure comprehensive, continuous, and equitable care for these individuals^(4,5)^.

Speech-language pathology, as an essential discipline in the interdisciplinary approach^(5)^ to children diagnosed with ASD, has advanced in the planning and implementation of interventions that include the necessary understanding of the individual's overall functioning, their family, caregivers, and educators. Speech-language pathologists have expanded their access to work in different settings^(6)^ that play a multifaceted role in the Brazilian healthcare system, such as Health Centers, Specialized Rehabilitation Centers (CER), Psychosocial Care Centers (CAPS), Community and Cooperative Centers (CECCO), Private Clinics, University Clinics, and Civil Society Organizations (OSC), among other possibilities. They may operate based on the concept of levels of complexity, depending on the services offered.

The interventions carried out in each of the aforementioned settings not only aim to develop the functional communication and social skills of these children, but also contribute to the process of expanding conditions for access to and permanence in social spaces, such as school and work, and other opportunities for development and autonomy. In working with autistic children, it is recommended^(4,5)^ that practices be related to the personalization of care - whether through assessment to identify delayed communication skills or for individualized therapeutic planning - without, however, disregarding the individual's family and educational context. In this process, the inclusion of family members^(4,5,7,8)^ in the therapeutic process should be carried out through guidance strategies to facilitate social engagement, communication, and interaction as fundamental elements for the success of the treatment.

In this regard, the work of the speech-language pathologist across different levels of healthcare complexity contributes both to mitigating the primary difficulties in speech and language development and to the understanding and construction of practices associated with the social processes adjacent to child development^(3-5,8)^. Thus, the practice of the speech-language pathologist at these various levels is shaped by the specific needs and demands of each individual, family, and context, aiming to promote communication health and the comprehensiveness^(9)^ of care.

In healthcare, especially during the early years of an individual's life^(5,8,9)^, the role of the speech-language pathologist is strategic for strengthening the comprehensiveness of care. This allows for the early detection of developmental alterations, the implementation of preventive measures, and the effective coordination of care at other levels of the health system^(6)^, regardless of the setting where these actions are performed. This integrated approach contributes to improving health indicators and promoting quality of life. Within this scope, the speech-language pathologist plays a fundamental role in care, health promotion, and the prevention of difficulties associated with or related to communication disorders. This includes conducting a situational diagnosis of the families and social environments that permeate the patient's life in order to promote educational actions, provide individual or group services (in daycare centers, schools, and community centers), and conduct home visits, among other possible forms of practice that can be developed by the professional.

Although the levels of complexity in healthcare are widely discussed within the scope of the settings and practices promoted by the Unified Health System (SUS)^(10)^, this understanding is not exclusive to public services. Pervading this conceptualization is the principle of comprehensive healthcare, which includes all measures aimed at human well-being, encompassing actions and services for promotion, prevention, treatment, and rehabilitation^(7)^ in various settings and services. This is considered in light of the complex underlying structure that constitutes the healthcare system in Brazil, and the equally complex collaborative dynamic between academic institutions, Civil Society Organizations (OSCs), and public services in health promotion^(11)^.

Given the growing prevalence^(12-14)^ of ASD worldwide and the complexity of its manifestations, it becomes imperative to understand how speech-language pathology has structured its therapeutic practices in different care contexts, which settings receive autistic children, their families, and caregivers, and what strategies permeate the praxis of speech-language pathology in this area of care. In view of the expanding training for speech-language pathologists, it is important that professionals are prepared to understand their practices as both being affected by and producing effects on social health policies. In doing so, they play a fundamental role in the way autism and other disorders are interpreted and addressed, reflecting the social views and perspectives on the autistic child and on autism itself.

In this vein, this study presents a systematic scoping review^(15)^ that seeks to analyze which practices are being reported by speech-language pathologists, and to characterize how these practices can be interpreted as methods for the inclusion and retention of autistic children in health services. Specifically, the objective of this scoping review is to identify and systematize the ways in which speech-language pathology practice for autistic children in health services is reported as an assessment and therapeutic strategy in Brazil.

## METHOD

This scoping review was conducted following the PRISMA-ScR^(15,16)^ guidelines to mitigate inconsistencies in data extraction and analysis. The review was carried out between March and July 2024. The review protocol was registered on the *OSF - Open Science Framework* ^(17)^ platform with the identifier 9Z2VN.

The review was guided by the formulation of a research question that informed the P (Population), C (Concept), C (Context) framework, in order to establish clear, significant objectives and eligibility criteria. The question was: What are the characteristics of speech-language pathology practice with autistic children in health services in Brazil? Population: published studies on children under 12 years of age with a diagnosis of autism spectrum disorder. Concept: speech-language pathology assessment and therapy strategies; Context: Brazil. Based on this definition, inclusion and exclusion criteria for eligible documents were established.

The inclusion criteria were as follows: documents that report and/or analyze types of speech-language pathology practices (therapeutic and assessment-based) conducted in Brazil with children and families in healthcare settings; studies that present research approval from a Research Ethics Committee, when applicable; and studies that describe the research design in a way that makes it possible to classify the study design and identify the population studied, the assessment and therapy strategies, and the context in which the study was conducted. Full-text studies written in Portuguese, English, or Spanish were included, with no limit on the publication date.

The exclusion criteria were as follows: documents reporting speech-language pathology practices and strategies exclusively with adolescents, adults, and/or older adults; research not conducted by speech-language pathologists; and documents that do not report and/or analyze types of therapeutic and assessment-based speech-language pathology practices. Studies conducted in countries other than Brazil were excluded, as were those not presenting approval from a Research Ethics Committee, when applicable. Also excluded were studies that did not describe the research strategy in a way that would make it possible to classify the study design and identify the study population, the assessment and therapy strategies, and the context in which the study was conducted.

### Ethical Considerations

This study comprises phase one of a research project in development, authorized by the Research Ethics Committee under CAAE number: 77227724.4.0000.5404.

### Search Strategy

Once the previous stages were completed, the descriptors were defined to develop the search syntax for the studies. The controlled vocabularies DeCS (Health Sciences Descriptors), MeSH (Medical Subject Headings), CINAHL Titles (Cumulative Index to Nursing and Allied Health Literature) from EBSCOhost, and Emtree were used to find descriptors with semantic characteristics that aligned with the research objectives. A preliminary search was conducted in PubMed to identify articles on the topic and refine the search terms, including terminological variations and relevant free terms. The databases and portals used for the review included: PubMed; Scopus; Web of Science; Embase; VHL-Bireme (BVS-Bireme); EBSCOhost; Proquest Central; Scielo; Google Scholar, and the Brazilian Digital Library of Theses and Dissertations (BDTD). The search strategy (Chart 1) was defined and executed by combining descriptors and free terms, and was adapted for each database (Chart 1).

**Chart 1:** Database Search Strategies Based on the Scoping Review Protocol Matrix^(17)^

**Objective**	To identify and systematize the ways in which speech-language pathology practice with autistic children in health services is reported as an assessment and therapeutic strategy in Brazil.				
**PCC**	**Population (P)**	**Concept (C)**	**Context (C)**		
**Limits**	**Autistic children, aged 2 to 12**	**Speech-language pathology assessment and therapy strategies**	**Brazil**		
**Construction and Combination**	**“Child”, “Child, Preschool”, “Autism Spectrum Disorder”, “Autistic Disorder”, “Asperger Syndrome”**	**Audiology, “Rehabilitative of Speech and Language Disorders”**	**“Speech, Language and Hearing Sciences”, “Speech Therapy”, Brazil OR Brasil Brazilian OR Brazilians**		
**Used**	(Child OR “Child, Preschool”) AND (“Autism Spectrum Disorder” OR “Autistic Disorder” OR “Asperger Syndrome”) AND (“Speech, Language and Hearing Sciences” OR Audiology OR “Rehabilitation of Speech and Language Disorders” OR “Speech Therapy”) AND (Brazil OR Brasil or brazilian or Brazilians)				
**Source**	**Strategy**			**# of Papers**	**Date**
**BDTD**	(Todos os campos: Criança OR “Pré-Escolar” E Todos os campos:”Transtorno do Espectro Autista” OR “Transtorno Autístico” OR “Síndrome de Asperger” E Todos os campos:Fonoaudiologia OR Audiologia OR “Reabilitação dos Transtornos da Fala e da Linguagem” OR Fonoterapia E Todos os campos:BRASIL OR BRASILEIROS OR BRASILEIRAS OR BRASILEIRO OR BRASILEIRA)			16	04/07/2024
**BVS**	((child OR children) OR (“Child, Preschool” OR “Preschool Child” OR “Preschool Children”)) AND ((“Autism Spectrum Disorder” OR “Autism Spectrum Disorders” OR “Autistic Spectrum Disorder” OR “Autistic Spectrum Disorders”) OR (“Autistic Disorder” OR “Kanner’s Syndrome” OR “Kanner Syndrome” OR “Infantile Autism” OR Autismo OR “Early Infantile Autism”) OR (“Asperger Syndrome” OR “Asperger Disease” OR “Asperger Disorder” OR “Asperger Disorders” OR “Asperger’s Disorder” OR “Aspergers Disorder” OR “Asperger’s Syndrome” OR “Aspergers Syndrome”)) AND ((“Speech, Language and Hearing Sciences”) OR (audiology) OR (“Rehabilitation of Speech and Language Disorders” OR “Language and Speech Disorder Rehabilitation” OR “Speech and Language Disorder Rehabilitation”) OR (“Speech Therapy” OR “Speech Therapies”)) AND ((Brazil OR Brasil OR brazilian OR brazilians))			**14**	**04/07/2024**
**EBSCOHOST Academic Search Premier 5 MEDLINE 3 MEDLINE Complete 3 CINAHL with Full Text 1 Computers & Applied Sciences Complete 1 ERIC 1 Library, Information Science & Technology Abstracts with Full Text 1**	(Child OR Children) OR (“Child, Preschool” OR “Preschool Child” OR “Preschool Children”) AND TI (“Autism Spectrum Disorder” OR “Autism Spectrum Disorders” OR “Autistic Spectrum Disorder” OR “Autistic Spectrum Disorders”) OR AB (“Autism Spectrum Disorder” OR “Autism Spectrum Disorders” OR “Autistic Spectrum Disorder” OR “Autistic Spectrum Disorders”) OR TI (“Autistic Disorder” OR “Kanner’s Syndrome” OR “Kanner Syndrome” OR “Infantile Autism” OR Autism OR “Early Infantile Autism”) OR AB (“Autistic Disorder” OR “Kanner’s Syndrome” OR “Kanner Syndrome” OR “Infantile Autism” OR Autism OR “Early Infantile Autism”) OR TI (“Asperger Syndrome” OR “Asperger Disease” OR “Asperger Disorder” OR “Asperger Disorders” OR “Asperger’s Disorder” OR “Aspergers Disorder” OR “Asperger’s Syndrome” OR “Aspergers Syndrome”) OR AB (“Asperger Syndrome” OR “Asperger Disease” OR “Asperger Disorder” OR “Asperger Disorders” OR “Asperger’s Disorder” OR “Aspergers Disorder” OR “Asperger’s Syndrome” OR “Aspergers Syndrome”) AND TI (“Speech, Language and Hearing Sciences”) OR AB (“Speech, Language and Hearing Sciences”) OR TI Audiology OR AB Audiology OR TI (“Rehabilitation of Speech and Language Disorders” OR “Language and Speech Disorder Rehabilitation” OR “Speech and Language Disorder Rehabilitation”) OR AB (“Rehabilitation of Speech and Language Disorders” OR “Language and Speech Disorder Rehabilitation” OR “Speech and Language Disorder Rehabilitation”) OR TI (“Speech Therapy” OR “Speech Therapies”) OR AB (“Speech Therapy” OR “Speech Therapies”) AND Brazil OR Brasil or brazilian or brazilians			09	04/07/2024
**EMBASE**	(‘child’ OR ‘children’ OR ‘child, preschool’ OR ‘preschool child’ OR ‘preschool children’) AND (‘autism spectrum disorder’:ti,ab,kw OR ‘autism spectrum disorders’:ti,ab,kw OR ‘autistic spectrum disorder’:ti,ab,kw OR ‘autistic spectrum disorders’:ti,ab,kw OR ‘autism’/exp OR ‘autistic disorder’:ti,ab,kw OR ‘kanner`s syndrome’:ti,ab,kw OR ‘kanner syndrome’:ti,ab,kw OR ‘infantile autism’:ti,ab,kw OR ‘autism’:ti,ab,kw OR ‘early infantile autism’:ti,ab,kw OR ‘asperger syndrome’/exp OR ‘asperger syndrome’:ti,ab,kw OR ‘asperger disease’:ti,ab,kw OR ‘asperger disorder’:ti,ab,kw OR ‘asperger disorders’:ti,ab,kw OR ‘asperger`s disorder’:ti,ab,kw OR ‘aspergers disorder’:ti,ab,kw OR ‘asperger`s syndrome’:ti,ab,kw OR ‘aspergers syndrome’:ti,ab,kw) AND (‘speech, language’ AND ‘hearing sciences’:ti,ab,kw OR ‘audiology’/exp OR ‘audiology’:ti,ab,kw OR (‘rehabilitation of speech’ AND ‘language disability’/exp) OR (((‘rehabilitation of speech’ AND ‘language disorders’:ti,ab,kw OR ‘language’) AND ‘speech disorder rehabilitation’:ti,ab,kw OR ‘speech’) AND ‘language disorder rehabilitation’:ti,ab,kw) OR ‘speech therapy’/exp OR ‘speech therapy’:ti,ab,kw OR ‘speech therapies’:ti,ab,kw) AND (‘brazil’ OR ‘brasil’ OR ‘brazilian’ OR ‘brazilians’)			35	04/07/2024
**Google Scholar**	(“Autismo” OR “Transtorno do Espectro Autista” OR TEA OR “Transtorno Autístico” OR “Síndrome de Asperger”) AND Fonoaudiologia AND BRASIL			47	04/07/2024
**PROQUEST CENTRAL**	((Child OR Children) OR (“Child, Preschool” OR “Preschool Child” OR “Preschool Children”)) AND (abstract(“Autism Spectrum Disorder” OR “Autism Spectrum Disorders” OR “Autistic Spectrum Disorder” OR “Autistic Spectrum Disorders”) OR title(“Autism Spectrum Disorder” OR “Autism Spectrum Disorders” OR “Autistic Spectrum Disorder” OR “Autistic Spectrum Disorders”) OR abstract(“Autistic Disorder” OR “Kanner’s Syndrome” OR “Kanner Syndrome” OR “Infantile Autism” OR Autism OR “Early Infantile Autism”) OR title(“Autistic Disorder” OR “Kanner’s Syndrome” OR “Kanner Syndrome” OR “Infantile Autism” OR Autism OR “Early Infantile Autism”) OR abstract(“Asperger Syndrome” OR “Asperger Disease” OR “Asperger Disorder” OR “Asperger Disorders” OR “Asperger’s Disorder” OR “Aspergers Disorder” OR “Asperger’s Syndrome” OR “Aspergers Syndrome”) OR title(“Asperger Syndrome” OR “Asperger Disease” OR “Asperger Disorder” OR “Asperger Disorders” OR “Asperger’s Disorder” OR “Aspergers Disorder” OR “Asperger’s Syndrome” OR “Aspergers Syndrome”)) AND (abstract(“Speech, Language and Hearing Sciences”) OR title(“Speech, Language and Hearing Sciences”) OR abstract(Audiology) OR title(Audiology) OR abstract(“Rehabilitation of Speech and Language Disorders” OR “Language and Speech Disorder Rehabilitation” OR “Speech and Language Disorder Rehabilitation”) OR title(“Rehabilitation of Speech and Language Disorders” OR “Language and Speech Disorder Rehabilitation” OR “Speech and Language Disorder Rehabilitation”) OR abstract(“Speech Therapy” OR “Speech Therapies”) OR title(“Speech Therapy” OR “Speech Therapies”)) AND (Brazil OR Brasil OR brazilian OR brazilians) https://www.proquest.com/search/2656553?accountid=8113			14	04/07/2024
**PUBMED**	(((((Child OR Children)) OR (“Child, Preschool” OR “Preschool Child” OR “Preschool Children”)) AND ((((((Autism Spectrum Disorder[MeSH Terms]) OR (“Autism Spectrum Disorder”[Title/Abstract] OR “Autism Spectrum Disorders”[Title/Abstract] OR “Autistic Spectrum Disorder”[Title/Abstract] OR “Autistic Spectrum Disorders”[Title/Abstract])) OR (Autistic Disorder[MeSH Terms])) OR (“Autistic Disorder”[Title/Abstract] OR “Kanner’s Syndrome”[Title/Abstract] OR “Kanner Syndrome”[Title/Abstract] OR “Infantile Autism”[Title/Abstract] OR Autism[Title/Abstract] OR “Early Infantile Autism”[Title/Abstract])) OR (Asperger Syndrome[MeSH Terms])) OR (“Asperger Syndrome”[Title/Abstract] OR “Asperger Disease”[Title/Abstract] OR “Asperger Disorder”[Title/Abstract] OR “Asperger Disorders”[Title/Abstract] OR “Asperger’s Disorder”[Title/Abstract] OR “Aspergers Disorder”[Title/Abstract] OR “Asperger’s Syndrome”[Title/Abstract] OR “Aspergers Syndrome”[Title/Abstract]))) AND (((((((“Speech, Language and Hearing Sciences”[Title/Abstract]) OR (Audiology[MeSH Terms])) OR (Audiology[Title/Abstract])) OR (Rehabilitation of Speech and Language Disorders[MeSH Terms])) OR (“Rehabilitation of Speech and Language Disorders”[Title/Abstract] OR “Language and Speech Disorder Rehabilitation”[Title/Abstract] OR “Speech and Language Disorder Rehabilitation”[Title/Abstract])) OR (Speech Therapy[MeSH Terms])) OR (“Speech Therapy”[Title/Abstract] OR “Speech Therapies”[Title/Abstract]))) AND (Brazil OR Brasil or brazilian or brazilians)			15	04/07/2024
**SCIELO**	(Criança OR Child OR Niño OR “Pré-Escolar” OR “Child, Preschool” OR Preescolar) AND (“Transtorno do Espectro Autista” OR “Autism Spectrum Disorder” OR “Trastorno del Espectro Autista” OR “Transtorno Autístico” OR “Autistic Disorder” OR “Trastorno Autístico” OR “Síndrome de Asperger” OR “Asperger Syndrome” OR “Síndrome de Asperger”) AND (Fonoaudiologia OR “Speech, Language and Hearing Sciences” OR Fonoaudiología OR Audiologia OR Audiology OR Audiología OR “Reabilitação dos Transtornos da Fala e da Linguagem” OR “Rehabilitation of Speech and Language Disorders” OR “Rehabilitación de los Trastornos del Habla y del Lenguaje” OR Fonoterapia OR “Speech Therapy” OR Logopedia) AND (Brasil OR Brazil OR brazilian or brazilians)			**03**	**04/07/2024**
**SCOPUS**	(((ALL (child OR children) OR ALL (“Child, Preschool” OR “Preschool Child” OR “Preschool Children”))) AND ((TITLE-ABS-KEY (“Autism Spectrum Disorder” OR “Autism Spectrum Disorders” OR “Autistic Spectrum Disorder” OR “Autistic Spectrum Disorders”) OR TITLE-ABS-KEY (“Autistic Disorder” OR “Kanner’s Syndrome” OR “Kanner Syndrome” OR “Infantile Autism” OR utismo OR “Early Infantile Autism”) OR TITLE-ABS-KEY (“Asperger Syndrome” OR “Asperger Disease” OR “Asperger Disorder” OR “Asperger Disorders” OR “Asperger’s Disorder” OR “Aspergers Disorder” OR “Asperger’s Syndrome” OR “Aspergers Syndrome”))) AND ((TITLE-ABS-KEY (“Speech, Language and Hearing Sciences”) OR TITLE-ABS-KEY (audiology) OR TITLE-ABS-KEY (“Rehabilitation of Speech and Language Disorders” OR “Language and Speech Disorder Rehabilitation” OR “Speech and Language Disorder Rehabilitation”) OR TITLE-ABS-KEY (“Speech Therapy” OR “Speech Therapies”)))) AND (ALL (brazil OR brasil OR brazilian OR brazilians))			**68**	**04/07/2024**
**WEB OF SCIENCE**	Child OR Children (Topic) or “Child, Preschool” OR “Preschool Child” OR “Preschool Children” (Topic) and Preprint Citation Index (Exclude – Database) AND”Autism Spectrum Disorder” OR “Autism Spectrum Disorders” OR “Autistic Spectrum Disorder” OR “Autistic Spectrum Disorders” (Topic) or “Autistic Disorder” OR “Kanner’s Syndrome” OR “Kanner Syndrome” OR “Infantile Autism” OR Autism OR “Early Infantile Autism” (Topic) or “Asperger Syndrome” OR “Asperger Disease” OR “Asperger Disorder” OR “Asperger Disorders” OR “Asperger’s Disorder” OR “Aspergers Disorder” OR “Asperger’s Syndrome” OR “Aspergers Syndrome” (Topic) and Preprint Citation Index (Exclude – Database) AND “Speech, Language and Hearing Sciences” (Topic) or Audiology (Topic) or “Rehabilitation of Speech and Language Disorders” OR “Language and Speech Disorder Rehabilitation” OR “Speech and Language Disorder Rehabilitation” (Topic) or “Speech Therapy” OR “Speech Therapies” (Topic) and Preprint Citation Index (Exclude – Database) AND Brazil OR Brasil or brazilian or brazilians (Topic) and Preprint Citation Index (Exclude – Database) https://www.webofscience.com/wos/alldb/summary/15f85a45-0b96-4f98-a3e9-85b2b8fa44a5-f9012fe9/relevance/1			**12**	**04/07/2024**
**TOTAL**				**233**	
**TOTAL DUPLICATE REFERENCES**	79 DUPLICATE PAPERS DELETED IN RAYYAN 06 DUPLICATE PAPERS DELETED AFTER FINAL READING (other language)			**79+06=** **85**	
**TOTAL AFTER DUPLICATE DELETION**				**148**	

### Study selection

The following stage was carried out using the Rayyan^(18)^ software, into which the retrieved article files were imported for the removal of duplicates and subsequently screened by title and abstract. The selection and the decision for inclusion were performed by two researchers, independently and blindly, regarding the relevance of the articles for selection and inclusion in the study. Conflicts, when they arose, were resolved through the analysis of a third reviewer who determined the inclusion or exclusion of the documents. All documents available in full text that met the criteria established by the PRISMA^(16)^ protocol were included.

### Data extraction, mapping, and presentation of results

Data were obtained as specified in a previously developed extraction matrix^(17)^ in order to record the author, type and year of publication, study objective and population sample, concept of autism, type of intervention, speech-language pathology strategies used, and the research setting. The data were analyzed quantitatively via descriptive analysis using measures of central tendency and variability. The chi-square test, at a 5% significance level, was used to verify the relationship between categorical variables. All analyses and graphs were generated using R software^(19)^. The data extraction also involved a qualitative identification process based on the extraction matrix^(17)^ presented in the review protocol.

## RESULTS

**Figure 1:**
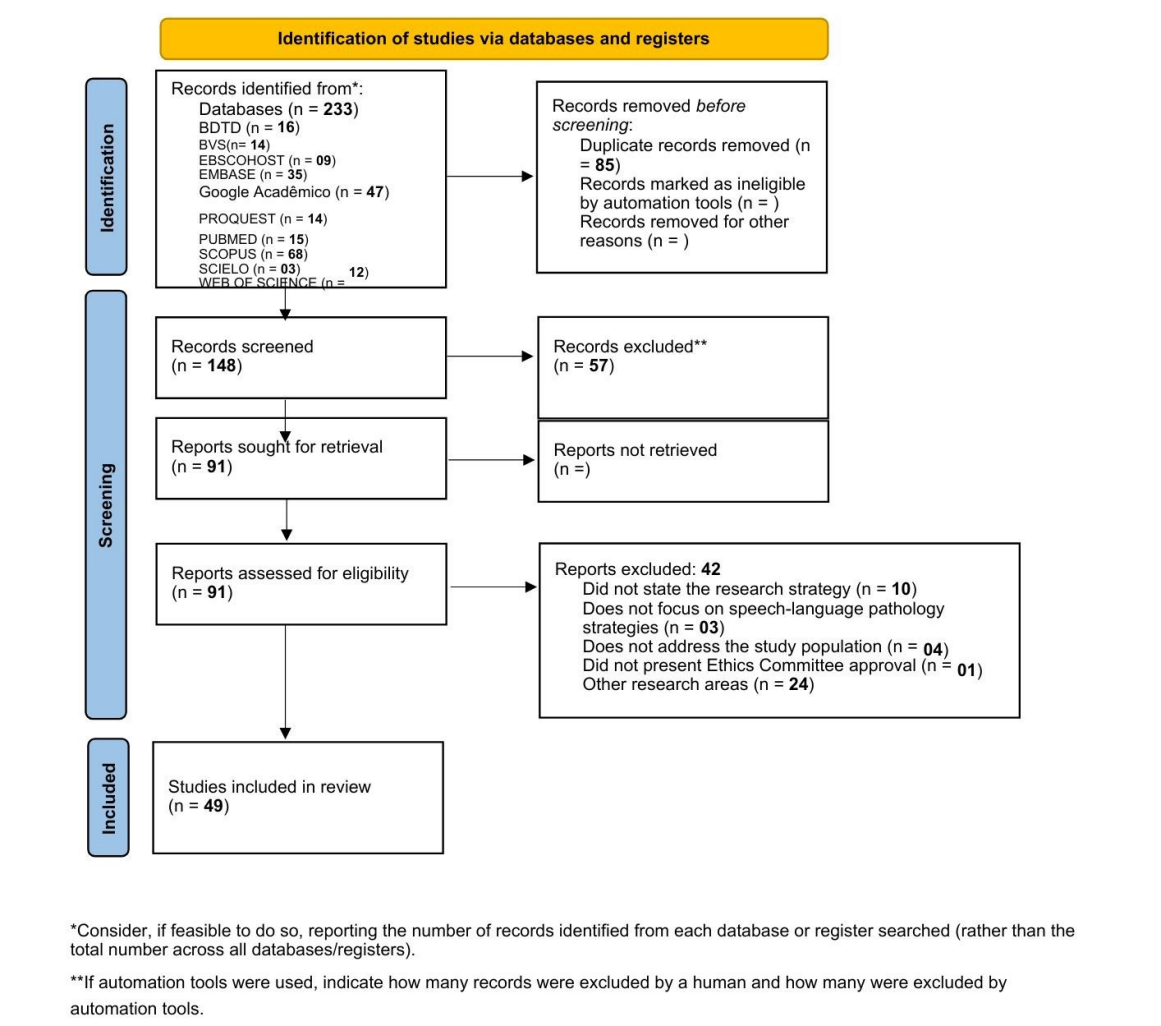
PRISMA-ScR 2020^(16)^ flow diagram, completed in accordance with the research results

The searches yielded 233 records. After the selection of records and removal of duplicates, 149 full-text studies were read, from which 92 were deemed eligible for analysis. An additional 42 records were excluded, resulting in 49 studies included in the review for data extraction (Figure 1)^(16)^.

The analyzed publications and the variables selected for analysis are presented in Chart 2. The scoping review reveals that the reviewed studies primarily focus on evaluating the efficacy of speech-language pathology interventions on the communication and language development of children with Autism Spectrum Disorder (ASD). The predominance of this type of research reflects the researchers' concern with validating specific therapeutic approaches, as well as understanding the impacts of the strategies used on the communicative engagement and language functionality of autistic children. Additionally, a significant number of studies seek to identify the communicative profile of these individuals, emphasizing both linguistic and pragmatic characteristics and caregivers' perceptions of the progress achieved throughout the interventions.

**Chart 2:** Study data populated from the scoping review protocol matrix to investigate the characteristics of interventions and speech-language pathology strategies in the care of autistic children in health services

Reference	Publication type	Publication year	Objective	Population and Sample	Autism Concept	Intervention type	Strategies	Intervention place	Conclusions
1. Balestro and Fernandes^(20)^	Paper	2018	To analyze the perceptions of caregivers of children with ASD regarding the functional communication profile of their children through the application of the Functional Communication Profile-Checklist (PFC-C).	CAREGIVERS = 62 children with ASD caregivers	DSM-IV	Therapeutic and Evaluative	Protocol and questionnaires	Teaching clinic	The hypothesis that it was possible to produce positive changes in the perspectives of parents of children with ASD regarding their children's communication through the administered questionnaire was confirmed.
Balestro and Fernandes^(21)^	Paper	2012	To develop and administer a questionnaire to survey the difficulties perceived by parents and/or caregivers of children with ASD in communicating with their children.	CAREGIVERS = 40 parents and/or children with ASD caregivers	Literature-based	Evaluative	Protocol and questionnaires	Teaching clinic	The administration of the developed questionnaire proved useful in identifying specific communication difficulties within the target population.
3. Balestro et al.^(22)^	Paper	2009	To demonstrate the efficiency and efficacy of an interactionist approach in speech-language therapy for 3 subjects with ASD.	CHILDREN = 3 individuals with ASD aged 6, 7 and 8.	Literature-based	Therapeutic and Evaluative	Play-based therapeutic context	Not informed	The cases analyzed demonstrated the efficacy of the interactionist approach in the subjects' language therapy.
4. Barbosa et al.^(23)^	Paper	2019	To identify the perceptions of parents of children with ASD regarding the speech-language pathology services within the multiprofessional team at the CAPSij.	CAREGIVERS = 9 parents of ASD children with ASD aged from 2 to 10.	Literature-based	Evaluative	Focus Group	CAPSij	In the parents' conversation, the effects of the clinical listening offered by the team, especially by the speech-language pathologist, were clear. Speech-Language Pathology was considered to be an integral and fundamental part of the mental health field when working with children with autism, which reinforces the value of the listening offered to parents by the team.
5. Botura et al.^(24)^	Paper	2021	To verify the existence of impairments in the pragmatic skills of Brazilian Portuguese-speaking children with ASD.	CHILDREN	DSM-V	Not applicable	Not applicable	Not applicable	The findings were inconclusive.
6. Campelo et al.^(25)^	Paper	2009	To analyze the verbal and non-verbal communication skills of autistic children using an analytical model based on communicative acts, identifying the communicative means and functions.	CHILDREN = 6 male individuals with ASD aged from 4 to 10.	DSM-IV	Evaluative	Play-based therapeutic context	Teaching clinic	More communicative acts occurred through gestural means as opposed to vocal and verbal means. There was a wide variety, with a predominance of non-focused, protest, exploratory, and reactive functions.
7. Cardoso and Montenegro^(26)^	Paper	2009	To determine and compare the functional communicative profiles of two groups of subjects with ASD.	CHILDREN = 8 children and adolescents with ASD aged from 3 to 17.	DSM-IV	Therapeutic and Evaluative	Play-based therapeutic context	Teaching clinic	Although a difference was identified between the group that underwent a change of therapist and the group that did not, this difference is not statistically significant, which can be attributed to the heterogeneity of the individuals and the small sample size.
8. Castro and Loureiro^(27)^	Paper	2020	An integrative literature review on music therapy and speech-language pathology regarding social interaction and language in children with ASD.	CHILDREN	DSM-IV	Not applicable	Not applicable	Not applicable	Fourteen studies were found that addressed the interfaces between speech-language pathology and music therapy in interventions for children with ASD regarding communication and social interaction. The results obtained were positive, suggesting that music promotes oral language expression, thereby improving the patient's prognosis.
9. Costa^(28)^	Dissertation [master]	2021	To investigate the language performance of preschool children diagnosed with ASD and compare it with that of children with typical language development through the application of the Brazilian version of the Preschool Language Assessment Instrument (PLAI-2).	CHILDREN = 15 Individuals with ASD aged from 3 to 5 years and 11 months.	DSM-V	Evaluative	Protocol and questionnaires	Teaching clinic	The sample group performed more poorly on the assessed skills compared to the comparison group.
10. Costa et al.^(29)^	Paper	2013	To translate into Brazilian Portuguese and culturally adapt the Children's Communication Checklist-2 (CCC-2), as well as to evaluate its internal consistency.	CAREGIVERS	Literature-based	Not applicable	Not applicable	Not applicable	The study presented the translation and cross-cultural adaptation process for the CCC-2. However, for the test to be widely used in clinical and research settings, validation of the Brazilian version of the instrument is still necessary.
11. Cruz and Gomes^(30)^	Undergraduate thesis	2020	To identify in the literature the main speech-language pathology interventions for children with ASD.	CHILDREN	DSM-V	Not applicable	Not applicable	Not applicable	It was found that through direct and indirect interventions, there is greater progress in the developmental milestones of these children. This validates that parent training is essential in the therapeutic process for children with ASD, as it promotes family involvement.
12. Cruz and Tamanaha^(31)^	Paper	2021	To analyze interactions involving the participation of nonverbal autistic subjects, grounded in a multimodal approach to human interaction.	CHILDREN = 2 nonverbal individual with ASD aged 7	DSM-V	Therapeutic and Evaluative	PECS Multimodal approach	Not informed	The analyst's attention to what occurs corporeally is crucial for grasping the way autistic children orient themselves to the complex and dynamic interactional ecology.
13. Netrval^(32)^	Thesis [doctorate]	2014	To develop a model of quality indicators for the management of Speech-Language Pathology services offered in institutions that serve individuals with ASD.	CHILDREN	DSM-V	Evaluative	Protocol and questionnaires	Multicenter	The administered questionnaire made it possible to learn more about a group of individuals with ASD receiving care in the municipality of São Paulo and the profile of the locations that offer this assistance. The collected data demonstrate that individualized therapeutic planning is still underutilized and that professionals rarely discuss the management of this care.
14. Ferreira et al.^(33)^	Paper	2023	To describe early intervention programs for ASD, analyzing and correlating the multi/interprofessional team, the role of Speech-Language Pathology, and family participation in care.	CHILDREN AND CAREGIVERS	DSM-V	Not applicable	Not applicable	Not applicable	Specialized and early intervention for children with ASD and their families has not established a single methodological standard, as a variety of therapeutic approaches exist in different countries, despite the predominance of the Early Start Denver Model.
15. Santos et al.^(34)^	Paper	2020	To analyze the impact of implementing the Picture Exchange Communication System (PECS) on the comprehension of instructions in children with ASD.	CHILDREN = 20 individuals with ASD aged from 6 to 12.	DSM-V	Therapeutic and Evaluative	PECS Multimodal approach	Teaching clinic	The hypothesis that the use of PECS has a positive effect on the comprehension of instructions was confirmed, as it provided the children with greater communicative and social engagement.
16. Deliberato et al.^(35)^	Paper	2021	To describe adult mediation and the communicative skills of children with ASD in play-based situations, through storytelling.	CHILDREN = 2 male individuals with TEA aged 2 and 3.	Literature-based	Therapeutic and Evaluative	Play-based therapeutic context	Teaching clinic	The research showed that the use of storytelling can be an important avenue for children with ASD with regard to interaction and communication.
17. Donadio et al.^(36)^	Paper	2023	To measure how Prompts for Restructuring Oral Muscular Phonetic Targets (PROMPT) improves speech in children with ASD.	CHILDREN = 1 male individual with TEA aged 6.	DSM-V	Therapeutic and Evaluative	Prompt method	Teaching clinic	It was observed that the participant showed improvements in speech, mandibular, labiofacial, and lingual control.
18. Evangelista^(37)^	Undergraduate thesis	2018	To determine the importance of speech-language pathology in the care process for patients with ASD within the Mental Health Network.	CHILDREN	Literature-based	Not applicable	Not applicable	Not applicable	It was observed that there is a significant gap in publications that address the topic of mental health from the perspective of Speech-Language Pathology, and furthermore, that the integration of Speech-Language Pathology into this field appears to be incipient and fragmented.
19. Fernandes et al.^(38)^	Paper	2010	To investigate the effect of using computers and specific software in speech-language therapy for autistic children.	CHILDREN = 23 individuals with ASD aged from 3 and 12.	Literature-based	Therapeutic and Evaluative	Informatics games	Teaching clinic	The subjects showed different reactions to the proposal of using computer resources during speech-language therapy.
20. Fernandes et al.^(39)^	Paper	2008	To determine the existence of observable differences based on the characteristics of the functional communication profile and socio-cognitive performance of children and adolescents with ASD.	CHILDREN = 46 individuals with ASD aged from 7 to 9.	Literature-based	Therapeutic and Evaluative	Language workshop	Teaching clinic	The results did not indicate statistically significant differences based on the characteristics of the functional communication profile and socio-cognitive performance, although differences were observable.
21. Fernandes et al.^(40)^	Paper	2010	To investigate the effect of providing guidance to mothers of autistic children on the children's communication and language processes, communicative profile, and socio-cognitive performance; and to investigate the effect of this guidance on the way mothers observe their children's performance.	PARENT–CHILD DYAD = 26 mother-child with ASD dyads	Literature-based	Parental guidance	Guidance groups	Teaching clinic	It is hypothesized that systematized and specific guidance, provided for short periods of time and with opportunities for follow-up, can contribute to the autistic child's communicative environment and to family understanding.
22. Ferreira et al.^(41)^	Paper	2021	To analyze the impact of implementing PECS on the burden index of mothers of children with ASD.	PARENT–CHILD DYAD = 20 mother-child with ASD dyads.	DSM-V	Therapeutic and Evaluative	PECS Multimodal approach	Teaching clinic	There was a trend towards a reduction in maternal burden scores following the implementation of PECS.
23. Freitas et al.^(42)^	Paper	2021	To describe the communication skills of children with ASD, considering both clinical and family perspectives.	CAREGIVERS = 10 parents and 04 therapists.	DSM-V	Evaluative	Protocol and questionnaires	Teaching clinic	It was found that both parents and therapists reported deficits in the verbal as well as the non-verbal communication skills of children with ASD.
24. Gomes et al.^(43)^	Paper	2004	To verify whether the clinical behavior of auditory hypersensitivity, as reported in interviews with parents, caregivers, and therapists of autistic children, corresponds to audiological findings, and to determine whether other types of stimuli also lead to auditory hypersensitivity.	CHILDREN = 46 individuals with ASD aged from 5 to 20.	DSM-IV	Evaluative	Protocol and questionnaires	Teaching clinic	Behavioral responses to sounds are not associated with the hypersensitivity of the auditory pathways, but rather with processing difficulties in the cerebral cortex, involving systems that are generally impaired in ASD, such as the limbic system.
25. Kamita et al.^(44)^	Paper	2020	To identify and analyze the characteristic findings of Cortical Auditory Evoked Potentials (CAEP) in children and/or adolescents with ASD in comparison with their typically developing peers.	CHILDREN AND ADOLESCENTS	DSM-V	Not applicable	Not applicable	Not applicable	The ASD population may present varied responses to the components of the CAEP (Cortical Auditory Evoked Potentials) in comparison to typically developing individuals.
26. Kwee et al.^(45)^	Paper	2009	To apply the transdisciplinary assessment protocol based on the Treatment and Education of Autistic and related Communication handicapped Children (TEACCH) program in order to observe the development of and make necessary adjustments to the programs of six of its students.	CHILDREN = 6 individuals with ASD aged from 7 to 12.	Literature-based	Therapeutic and Evaluative	Behavioral	Teaching clinic	It was observed that all students in the program made positive progress in all areas and that, despite their individual complexities, the gains in and maintenance of acquired behaviors were achieved and stabilized.
27. Martins and Fernandes^(46)^	Paper	2013	To evaluate changes in the Functional Communication Profile (FCP) and Socio-Cognitive Performance (SCP) of children with Autism Spectrum Disorders (ASD) following two short periods of intervention.	CHILDREN = 21 individuals with ASD from 2 to 12.	DSM-V	Therapeutic and Evaluative	Short-term intervention	Teaching clinic	With the 12-week intervention structure, few changes were observed in the FCP (Functional Communication Profile) and SCP (Socio-Cognitive Performance); therefore, the study suggests that further research of a longer duration is needed.
28. Matas et al.^(47)^	Paper	2009	To describe the audiological and electrophysiological results of individuals with psychiatric disorders, screening for peripheral and/or central auditory disorders.	CHILDREN AND ADOLESCENTS = 40 individuals with ASD from 8 to 19.	DSM-IV	Evaluative	Protocol and questionnaires	Not informed	A high prevalence of auditory impairments was observed in children with psychiatric disorders, although in some analyses, no statistically significant difference was found when the sample group was compared with the control group.
29. Mayerle et al.^(48)^	Paper	2021	To analyze the Mismatch Negativity (MMN) (a brain response to auditory changes based on memory) in children and adolescents with ASD and compare them with a control group.	CAREGIVERS = 68 caregivers of children and adolescents with ASD.	DSM-V	Evaluative	Protocol and questionnaires	Teaching clinic	Children and adolescents with ASD show higher latency and amplitude values in the MMN compared to individuals in the control group.
30. Menezes and Perissinoto^(49)^	Paper	2008	To assess joint attention skills in subjects with ASD across different contexts and with different communication partners.	CHILDREN = 20 children with ASD.	DSM-IV	Therapeutic and Evaluative	Play-based therapeutic context	Teaching clinic	The assessment of joint attention in a play context was effective, and the adult's intervention was reflected in an increase in these behaviors in semi-directed and imitation situations.
31. Miilher and Fernandes^(50)^	Paper	2009	To analyze the progression of functional and grammatical aspects at three distinct time points: initial assessment, after six months of therapy, and after twelve months of therapy.	CHILDREN = 10 male children with ASD aged from 2 to 11.	DSM-IV	Therapeutic and Evaluative	Play-based therapeutic context	Teaching clinic	There is a relationship between grammatical and pragmatic performance.
32. Miilher and Fernandes^(51)^	Paper	2012	To compare the pragmatic profile of communicative initiations with the two-dimensional profile involving aspects of initiation and responsiveness.	CHILDREN = speech therapy recordings from 10 child patients with ASD.	DSM-IV	Therapeutic and Evaluative	Play-based therapeutic context	Teaching clinic	The results highlighted the need to consider the two-dimensional communication profile and emphasized the necessity of qualifying the responses in order to differentiate the child's communicative skills.
33. Misquiatti et al.^(52)^	Paper	2014	To analyze the socio-cognitive performance of children and adolescents with ASD in two language therapy settings that differ in their physical structure.	CHILDREN AND ADOLESCENTS = 10 children and adolescents with ASD aged from 4 to 13.	Literature-based	Therapeutic and Evaluative	Play-based therapeutic context	Teaching clinic	The creation of pre-established physical environments or specific materials is not and should not be considered indispensable for language therapy.
34. Misquiatti et al.^(53)^	Paper	2015	To assess the burden of family caregivers of children with ASD, according to the caregivers' own perceptions.	CAREGIVERS = 10 family members of children with ASD, and 10 family members of children with language disorders.	DSM-IV	Evaluative	Protocol and questionnaires	Not informed	The observed results showed no difference in the mean burden index between family caregivers of children with ASD and those of children with language disorders.
35. Misquiatti and Fernandes^(54)^	Paper	2011	To analyze the functional communicative profile of children and adolescents with ASD in two different language therapy settings, with the differences concentrated in the physical aspect of the environment.	CHILDREN = 10 children and adolescents with ASD from 4 to 13.	Literature-based	Therapeutic and Evaluative	Environmental enrichment	Teaching clinic	The physical environment studied did not have a significant influence on the functional communicative profile of the autistic subjects, although there were individuals who showed a tendency to perform better in one environment compared to the other.
36. Moraes^(55)^	Dissertation [master]	2019	To verify the effectiveness of a remote stimulation program implemented at LIFDEA-FMUSP.	CHILDREN AND ADOLESCENTS = 30 children and adolescents with ASD aged from 6 to 16.	DSM-V	Therapeutic and Evaluative	Teletherapy	Teaching clinic	It was found that the number of strategies proposed for a single therapeutic intervention objective can vary, revealing the wide variety of possibilities for addressing the characteristics of autism spectrum disorder.
37. Neubauer and Fernandes^(56)^	Paper	2013	To evaluate the use of a checklist, as a substitute for the full protocol, for analyzing the Functional Communication Profile (FCP).	CHILDREN = video recordings of speech therapy sessions with 50 children with ASD aged 3 to 12 years.	DSM-IV	Evaluative	Play-based therapeutic context	Teaching clinic	The checklist can be used as an instrument to monitor the therapeutic processes of children with ASD, but it does not replace the complete instrument.
38. Navarro^(57)^	Paper	2018	To reinterpret speech-language pathology practice in the context of Equine-Assisted Therapy based on Discursive Neurolinguistics (DN).	CHILDREN = 1 male child with ASD.	DSM-V	Therapeutic and Evaluative	Equine-assisted therapy	Not informed	The reinterpretation of speech-language pathology services in the context of Equine-Assisted Therapy, as made possible by Discursive Neurolinguistics (DN), expands upon and explains the events of the equine-assisted therapy setting as a whole.
39. Pereira et al.^(58)^	Paper	2019	To investigate the effects of speech-language pathology intervention with Augmentative and Alternative Communication (AAC) on the communicative acts of children with ASD.	CHILDREN = 3 male children with ASD aged from 2 e 4.	DSM-V	Therapeutic and Evaluative	PECS Multimodal approach	Teaching clinic	The use of AAC (Augmentative and Alternative Communication) in speech-language pathology clinical practice is proving to be promising and effective in promoting the development of communication skills in individuals with ASD.
40. Roncati et al.^(59)^	Paper	2019	To replicate the procedures of Coon and Miguel (reinforcement histories with a specific prompt topography) with children with ASD, who are commonly exposed to tact and echoic prompts when learning verbal behavior.	CHILDREN = 3 male children with ASD aged 3, 6 and 7.	Literature-based	Therapeutic and Evaluative	Prompt method	Not informed	For professionals who plan lessons to teach intraverbal behavior to children with ASD, information about a patient's history with a specific type of prompt can be essential.
41. Rosa^(60)^	Dissertation [master]	2022	To investigate oral story narrative performance in children and adolescents with ASD without intellectual disability (ID).	CHILDREN = 27 male individuals with ASD and without Intellectual disability.	Literature-based	Evaluative	Protocol and questionnaires	Teaching clinic	The sample group achieved performance similar to that of individuals with typical language development in oral story comprehension and superior performance in oral story narrative production.
42. Santos et al.^(61)^	Paper	2022	To investigate the contribution of teletherapy to the development of communication skills in children with ASD during the COVID-19 pandemic.	CHILDREN = 8 children with ASD aged from 2 to 8.	DSM-V	Evaluative	Teletherapy	Not informed	The results demonstrated that speech-language teleconsultation for children with ASD during the social isolation resulting from the COVID-19 pandemic proved to be a viable option that produced advancements in the children's communication.
43. Segeren and Fernandes^(62)^	Paper	2019	To investigate the clinical case profile of a referral health service.	CHILDREN = Medical records of patients who underwent speech-language therapy at LIF-DEA.	DSM-V	Not applicable	Not applicable	Teaching clinic	The study showed the need for systematic records of the work performed, preferably with computerized databases. A limitation in data characterization and a reduction in data loss were observed following digitization.
44. Silva et al.^(63)^	Paper	2007	To describe the speech-language pathology intervention process for a child diagnosed with autism.	CHILDREN = a 2 year-old boy with ASD.	DSM-IV	Therapeutic and Evaluative	Play-based therapeutic context	Teaching clinic	The therapeutic strategies presented here proved to be effective for the child in question, as she showed progress throughout the speech-language therapy process.
45. Sun et al.^(64)^	Paper	2017	To identify effective methods for stimulating language and communication in children with ASD, which is fundamental for the effective use of available resources to support these children.	CHILDREN = 20 children with ASD aged from 5 to 12.	Literature-based	Therapeutic and Evaluative	Play-based therapeutic context	Teaching clinic	Intervention that includes activities for stimulating executive functions in children with ASD requires further investigation.
46. Tamanaha and Perissinoto^(65)^	Paper	2011	To analyze and compare the scope and rate of the developmental process in children with ASD receiving combined direct and indirect speech-language pathology intervention versus those receiving only indirect intervention.	CHILDREN = 11 male children with ASD and Asperger Syndrome aged from 4 to 10	DSM-IV	Therapeutic and Evaluative	Play-based therapeutic context	Teaching clinic	The trend of better performance among the children receiving both interventions showed that the combination of direct and indirect actions is fundamental to the speech-language therapy process for children with ASD.
47. Tamanaha et al.^(66)^	Paper	2008	To evaluate the developmental progress of autistic children in the context of direct and indirect intervention, based on mothers' responses to the Autism Behavior Checklist.	CAREGIVERS = 11 mothers of children with ASD and Asperger Syndrome.	DSM-IV	Therapeutic and Evaluative	Play-based therapeutic context	Teaching clinic	Mothers in both the direct therapy group (GT) and the indirect group (GO) observed behavioral changes. The trend of better scores in the GT was likely due to the efficacy of the direct intervention and not to a lack of attention from the parents in the GO.
48. Tamanaha et al.^(67)^	Paper	2015	To evaluate the efficacy of speech-language therapy intervention for children with Autism Spectrum Disorders.	CHILDREN = 11 children with ASD and mild to moderate intellectual disability.	DSM-V	Therapeutic and Evaluative	Protocol and questionnaires	Teaching clinic	It was concluded that the combination of direct and indirect interventions is fundamental for the improved performance of these children.
49. Santos^(68)^	Thesis [doctorate]	2017	To evaluate the effectiveness of several instruments (PFC-C, FCP-Rr - TP, FCP-Rr - OF, PFC, ToPS) for investigating the pragmatic skills of children with ASD.	CHILDREN = 30 children with ASD	DSM-V	Therapeutic and Evaluative	Protocol and questionnaires	Teaching clinic	The FCP-Rr and PFC-C protocols were sensitive and consistent in assessing the pragmatic skills of individuals with autism based on therapists' responses.

The relationship between the studies' objectives and their conclusions reinforces the efficacy of various therapeutic strategies. Interventions such as the Picture Exchange Communication System (PECS) and music therapy were reported as tools that promote communicative engagement, leading to advancements in language development and social interaction. Furthermore, the studies indicate that interactive approaches and play-based therapeutic contexts play a central role in improving the children's communication skills. Also noted, although reported in a smaller number of publications, was the importance of parental guidance and clinical listening. This was evidenced by research that analyzed the impact of interventions on the daily lives of families, highlighting the need for active caregiver involvement in the therapeutic process.

Regarding “population and sample”, the studies predominantly involved children diagnosed with ASD between the ages of 02 and 12. However, some studies also included adolescents and young adults (Chart 2). In some cases, the children's caregivers were also analyzed, particularly when the research focus was on observing the impact of family guidance on the therapeutic process. Furthermore, the comparison between children and adolescents, as observed in some studies, allowed for a broader perspective on the progression of linguistic skills throughout language and communication development.

Regarding the detailed data (Table 1), it was found that of the reviewed publications, 85% are articles^(20-27,29,31,33-36,38-54,56-59,61-67)^; 6.1% are master's theses^(28,55,60)^; 4.1% are doctoral dissertations^(32,68)^; and 4.1% are undergraduate final papers^(30,37)^. The majority of the works, 65.3%, were published between 2013 and 2024^(20,23,24,27,29,31,33-59,61,62,64,67,68)^ (Table 1), coinciding with the publication period of the updated Diagnostic and Statistical Manual of Mental Disorders (DSM-5), while 34.7% were published between 2004 and 2012^(20,21,25,26,38-40,43,45,47,49-51,54,63,65,66)^.

**Table 1:** Frequency distribution of “Publication Type”, “Concept of Autism”, “Intervention Type”, “Intervention Location”, and “Intervention Strategies”

	Absolute frequency	Percentage Frequency
**Publication type**		
Papers	42	85.7%
Dissertation [Master]	3	6.1%
Thesis [Doctorate]	2	4.1%
Undergraduate Thesis	2	4.1%
**Concept of autism**		
DSM-V	20	40.8%
Literature based	15	30.6%
DSM-IV	14	28.6%
**Intervention type**		
Therapeutic and Evaluative	27	67.5%
Evaluative	12	30.0%
Parental guidance	1	2.5%
**Intervention location**		
Teaching clinic	33	78.6%
Not reported	7	16.7%
CAPSij	1	2.4%
Multicenter	1	2.4%
**Intervention strategies**		
Play-based therapeutic context	12	30.0%
Protocol and questionnaires	12	30.0%
PECS Multimodal approach	4	10.0%
Prompt method	2	5.0%
Teletherapy	2	5.0%
Behavioral	1	2.5%
Environmental enrichment	1	2.5%
Equine-assisted therapy	1	2.5%
Focus Group	1	2.5%
Guidance groups	1	2.5%
Short-term intervention	1	2.5%
Informatic games	1	2.5%
Language workshop	1	2.5%

The data extraction results revealed a relationship between the studies published by speech-language pathologists in Brazil and the publication and update periods of the DSM. The DSM-5 is cited in 40.8% of the publications^(24,28,30,34,41,42,44,46,48,55,57,58,61,62,67,68)^, the DSM-IV in 28.6%^(20,25-27,43,47,49-51,53,56,63,65,66)^, and 30.6% cite these manuals indirectly by using other publications (literature-based) to describe the symptoms of autism^(20-23,29,35,37-40)^. The chi-square test for independence indicates that the concept of autism used is dependent on the study's publication date (p-value < 0.001) (Table 2). Between 2004 and 2012, the concept of autism in the research formulation was derived primarily from the DSM-IV, whereas from 2013 to 2024, it shifted to the DSM-5. Concepts based on existing literature (literature-based) were used similarly across both periods.

**Table 2:** Contingency Table (Cross-Tabulations) between: “Intervention Type” vs. “Intervention Location”, “Publication Year” vs. “Concept of Autism”, and “Intervention Types” vs. “Intervention Strategies”, highlighting the p-value of the test for independence

Intervention type	CAPSij	Teaching clinic	Not reported	p-value
Evaluative	1	8	3	
Parental guidance	0	1	0	0.7142
Therapeutic and Evaluative	0	23	4	
Concept of autism	2004-2012	2013-2024		
Literature-based	7	8		
DSM-IV	10	4		<0,001
DSM-V	0	20		
Intervention strategies	Evaluative	Parental guidance	Therapeutic and Evaluative	
PECS Multimodal approach	0	0	4	
Behavioral	0	0	1	
Play-based therapeutic context	1	0	11	
Environmental enrichment	0	0	1	
Equine-assisted therapy	0	0	1	
Focus Group	1	0	0	
Guidance groups	0	1	0	<0.01
Short-term intervention	0	0	1	
Informatics games	0	0	1	
Prompt method	0	0	2	
Language workshop	0	0	1	
Protocol and questionnaires	8	0	3	
Teletherapy	1	0	1	

Of the studies reviewed, the predominant “Intervention type” was the “therapeutic and evaluative” approach, highlighting a focus on the treatment and measurement of the children's progress. For this analysis, publications aiming to conduct an evaluative survey and/or analysis and associating them with therapeutic practices were included in the “therapeutic and evaluative” category; publications focusing solely on assessment procedures were included in the “evaluative” category; and publications reporting practices exclusively with parents and/or caregivers, excluding assessment practices, were included in the “parental guidance” category. Based on this, it was found that the majority of publications, 67.5%^(20,22,26,31,34-36,38-40,45,46,49-52,54,55,57-59,63-68)^, featured a “type of intervention” associated with therapeutic and evaluative practices. In contrast, 30%^(2,23,25,28,42,43,47,48,53,56,60,61)^ reported solely evaluative practices, and only 2.5%^(69)^ presented practices developed with parental guidance (Table 1).

In the “intervention location” category, 78.6% of the publications^(20,21,25,26,28,34-36,38-43,45,46,48-52,54,55,57,58,60,62-68)^ report practices conducted in the teaching clinic of public and private universities; 2.4% were conducted in a CAPSij (Psychosocial Care Center for Children and Adolescents)^(23)^; 2.4% report multi-center studies^(32)^; and 16.7% identify the practice location as a “health service” but without further specification^(24,27,29-31,33,35,44,47,53,57,59,61)^. In other words, the vast majority of publications report practices associated with assessment or rehabilitation activities, which are predominantly carried out in university clinics.

Regarding the comparison of the collected data (Table 2), the independence chi-square test indicates that the intervention location does not depend on the type of intervention performed (p=.7142). That is, regardless of the intervention location, the most frequent type of intervention is “Therapeutic and Evaluative”. Conversely, the university clinic is the most frequent location, regardless of the type of intervention.

The data extracted on the “intervention strategies” employed in the studies varied widely, including standardized protocols, questionnaires, focus groups, language workshops, and play-based methods. There were also reports of interactive approaches, such as the use of narrative stories and computer games. Studies that investigated the influence of the therapeutic environment indicated that the physical structure and organization of spaces can affect children's responsiveness and performance in communicative interactions. The most frequently reported “therapeutic strategy” in the publications was (Table 1) “play-based therapeutic context”^(22,25,26,35,49-52,63-66)^, with a frequency of 30%, equal to the use of “protocols and questionnaires”^(20,21,28,32,42,43,47,48,53,60,67,68)^. A “multimodal approach with the use of PECS”^(31,34,41,58)^ appeared in 10% of studies, followed by the “Prompt method”^(36,59)^ at 5%, and “teletherapy”^(55,61)^ also at 5%. The following strategies each accounted for 2.5% of the total: “behavioral approach”^(45)^, “environmental enrichment”^(54)^, “equine-assisted therapy”^(57)^, “focus group”^(23)^, “short-term intervention”^(46)^, “computer games”^(38)^, and “language workshop”^(39)^.

When the cross-analysis between “intervention types” and “strategies” was conducted (Table 2), p<.01 indicates that the therapeutic strategy depends on the prioritized type of intervention. When the intervention is solely “evaluative”, the preferred strategy is the use of “protocols and questionnaires”. However, when it is “therapeutic and evaluative”, the strategy is predominantly a “play-based therapeutic context”. Therapeutic strategies such as the play-based therapeutic context and the use of protocols and questionnaires are widely reported. It is observed that a play-based approach is preferred in therapeutic interventions, while standardized protocols predominate in studies focused exclusively on evaluation.

## DISCUSSION

The predominance of studies focused on evaluating the efficacy of speech-language pathology interventions reflects the researchers' concern with validating therapeutic approaches and understanding the impacts of the strategies used on the communicative engagement of autistic children. The concentration of these practices in university clinics suggests a tendency in the reviewed studies to focus on treatment and evaluation, to the detriment of health promotion actions in community contexts.

The transition from the DSM-IV to the DSM-5 as the predominant framework in autism research reflects the paradigmatic alignment of researchers in understanding the disorder within this scientific community. Although this transition was largely driven by biological, traditional, and deficit-focused perspectives^(70)^, it is important to consider that this approach does not exhaust the possibilities for understanding autism. Different theoretical and methodological approaches contribute to the construction of knowledge in the field, and the predominance of one model does not imply the exclusion of others.

The DSM^(71,72)^ was created to assist mental health professionals in diagnosing mental disorders. Its first edition, the DSM-I, was published in 1952 by the American Medico-Psychological Association, later renamed the American Psychiatric Association (APA). Since then, the manual has undergone four revisions: DSM-II, DSM-III, DSM-IV, and DSM-5. Over time, the understanding of autism has undergone transformations that have accompanied scientific advances, changes in social attitudes, and improvements in diagnostic methods^(70,73)^. Initially, autism was confused with other psychiatric conditions and was later recognized as a specific spectrum disorder. The history of the conceptualization of autism shows the difficulty in reaching a consensus^(74,75)^, but the DSM, a tool created by the APA, finds broad agreement among clinical professionals and researchers in Brazil. In the country, the identification of the autistic condition and the description of its symptoms are based on the definitions contained in this tool^(72)^.

Although the DSM is an essential tool for standardizing diagnosis and communication among professionals, it also receives criticism^(8,21,76-78)^ for its rigidity and tendency to neglect the subjectivity of autistic individuals. Furthermore, critics argue that its rigid criteria can neglect the diversity of autistic individuals. These authors report and support the view that autism is a variation of human neurodiversity, and not a disorder to be standardized or corrected. In this sense, excessive specialization and the broadening of diagnostic criteria could contribute to an “autism epidemic”. On the other hand, other researchers argue that maintaining the Diagnostic and Statistical Manual of Mental Disorders (DSM) is essential for the diagnosis and therapy of autism, as it provides a standardized classification system that facilitates communication among researchers, clinicians, and policymakers.

The DSM-5 brought advancements in diagnostic refinement by grouping autism spectrum disorders under a single diagnosis and emphasizing clinical criteria that include deficits in social communication and interaction, as well as restricted and repetitive patterns of behavior. However, this approach^(79-81)^, centered on medical and neurobiological criteria, can, in some contexts, limit the understanding of the social and phenomenological complexity and the lived experiences of autistic individuals - a perspective emphasized by social and neurodiversity approaches. Furthermore, the persistent use of concepts based on existing literature demonstrates the coexistence of different interpretative approaches. Research that utilizes interdisciplinary theoretical frameworks, such as sociocognitive, pragmatic, and inclusive education studies, points to the need to broaden the scope of investigation by incorporating perspectives that go beyond classification and diagnosis^(78)^. These approaches highlight, for example, the role of social context, alternative and augmentative communication, and interaction with environments in the developmental and learning processes of autistic children.

The use of diagnostic criteria defined by the APA may have directed the way the reviewed studies constructed their practices with children with autism. The descriptions report practices carried out in settings more related to rehabilitative care than to health promotion. A vast majority of the studies reveal practices conducted in university clinics. The predominance of “Therapeutic and Evaluative” interventions in settings such as university clinics reflects the tendency to concentrate actions on the treatment and evaluation of children with Autism Spectrum Disorder (ASD). In other words, the concentration of these practices in clinical and academic environments limits the scope of speech-language pathology actions, especially with regard to health promotion and prevention in community contexts.

In the same vein, the cross-analysis between “intervention types” and “strategies” demonstrated that the choice of therapeutic strategies depends on the type of intervention adopted (p<.01). Exclusively evaluative interventions tend to use structured protocols, whereas combined therapeutic interventions prefer interactive and play-based contexts. If, on the one hand, clinical speech-language pathology assessment^(79,80)^ is important for establishing a functional diagnosis to construct an intervention plan that meets the specific needs of each patient, on the other hand, health promotion, which also belongs in and should be carried out in clinical settings, aims to broaden the possibilities of care in the face of the different facets of individual and social life^(82)^. In the context of ASD, this would imply actions that promote social inclusion, public awareness, and support for families, aspects that can be underestimated when the focus falls predominantly on traditional settings, intervention types, and therapeutic strategies.

The analysis of the “conclusions” from the reviewed research suggests that some investigations failed to establish statistically significant differences between the evaluated groups, possibly due to sample heterogeneity and the small number of participants. Furthermore, there is an emphasis on studies conducted in university clinic settings, which limits the understanding of the interventions' effects in community health and family contexts. In many cases, the research suggests that more prolonged and comprehensive interventions would be necessary to capture subtle and sustainable changes in the communication of children with ASD.

Thus, the review of these conclusions points to the importance of broadening the focus of research in the field of speech-language pathology, particularly concerning the setting and “type of intervention”, to include the diversity of social and communicative contexts of children with ASD.

## CONCLUSION

This scoping review made it possible to identify and systematize speech-language pathology practices for autistic children in health services in Brazil, highlighting the predominance of studies describing therapeutic and evaluative approaches conducted primarily in university clinics. The analysis of the studies revealed that although speech-language pathology has advanced in personalizing care and adapting therapeutic strategies in healthcare settings - through the diversification of therapeutic approaches and a preference for play-based and contextual strategies - interventions centered on diagnosis and rehabilitation still predominate.

The diversity of strategies identified points to the possibility of extending these practices to consider not only the difficulties of the individuals receiving care but also the need to include practices that consider the individual's relationship with their family, educational, and social context.

However, it is important to emphasize that this study is limited by the search strategy employed in this review protocol. Given this, an expansion of research into community-based and multiprofessional practices is recommended, as are new studies that explore different levels of healthcare, allowing for a better understanding of speech-language pathology care in ASD. Strengthening the coordination between health, education, and social assistance should also be encouraged, promoting a more comprehensive care model that includes not only assessment and treatment but also the inclusion and promotion of the social quality of life for autistic children and their families.

The percentage-frequency predominance of studies conducted in university clinics points to an under-representation of practices developed in settings such as Basic Health Units (UBS), Community Centers, and Child Psychosocial Care Centers (CAPSij), limiting the view of the diversity of interventions applied in public services. This gap highlights the need to broaden methodological diversity and research settings by incorporating investigations that analyze the effectiveness of therapeutic strategies in different realities and throughout the children's development.

The study suggests that reports of speech-language pathology practices in community health environments are scarce, creating a gap in the care of autistic children. The integration of interdisciplinary approaches, such as collaborative practices among speech-language pathologists, educators, and psychologists, can enhance the effectiveness of interventions and provide comprehensive support for children and their families.
