# Supplementary material for: Intervention characteristics and speech therapy strategies in care for autistic children in health services: a scoping review
Source: Codas. 2025 Dec 8;37(6):e20250012. doi: 10.1590/2317-1782/e20250012en (PMC12704610; doi:10.1590/2317-1782/e20250012en)
Supplement: Translation

# Translation: Características de intervenção e estratégias fonoaudiológicas na atenção à criança autista em serviços de saúde: revisão de escopo

**DOI:** 10.1590/2317-1782/e20250012pt

**Authors:** Renata Chrystina Bianchi de Barros, Eric Batista Ferreira, Priscila Mara Ventura Amorim Silva, Mariana Minante Khalil, Ana Paula de Moraes Oliveira

**Affiliations:** 1 Departamento de Desenvolvimento Humano e Reabilitação, Faculdade de Ciências Médicas, Universidade Estadual de Campinas – UNICAMP - Campinas (SP), Brasil.; 2 Departamento de Estatística, Universidade Federal de Alfenas – UNIFAL-MG – Alfenas (MG), Brasil.; 3 Centro de Estudos e Pesquisas em Reabilitação “Prof. Dr. Gabriel O.S. Porto” – CEPRE, Universidade Estadual de Campinas – UNICAMP - Campinas (SP), Brasil.; 4 Faculdade de Medicina, Universidade Federal de Alfenas – UNIFAL-MG - Alfenas (MG), Brasil.; 5 Biblioteca, Universidade Estadual de Campinas – UNICAMP - Campinas (SP), Brasil.

**Keywords:** Autismo, Transtorno do Espectro Autista, Reabilitação dos Distúrbios da Fala e da Linguagem, Fonoaudiologia, Brasil

## Abstract

**Objetivo:**

Identificar e sistematizar a prática fonoaudiológica com crianças autistas em serviços de saúde relatadas enquanto estratégia avaliativa e terapêutica no Brasil.

**Estratégia de pesquisa:**

Revisão do tipo escopo seguindo as diretrizes do Joanna Briggs Institute e PRISMA-ScR. Artigos, ensaios, revisões e literatura cinzenta disponíveis até 4 de julho de 2024 foram recuperados em bases como PubMed, Scielo, Scopus, Web of Science, ProQuest Central, Embase, EBSCOhost, BVS, BDTD e Google Acadêmico. Listas de referências e revisões sistemáticas relevantes também foram verificadas para documentos adicionais.

**Critérios de seleção:**

Baseados no formato PCC (Participantes: crianças autistas de 2 a 12 anos; Conceito: estratégias de avaliação e terapia fonoaudiológica; Contexto: Brasil).

**Análise dos dados:**

Os dados foram extraídos com matriz previamente elaborada considerando autor, tipo/ano de publicação, objetivo, amostra, conceito de autismo, tipo/estratégia de intervenção, local e conclusão. Foram realizadas análises quantitativas descritivas e qualitativas.

**Resultados:**

Foram incluídos 49 estudos na revisão, sendo possível identificar que as práticas fonoaudiológicas direcionadas a crianças autistas em serviços de saúde brasileiros têm predominância de abordagens terapêuticas e avaliativas realizadas principalmente em clínicas-escola.

**Conclusão:**

Embora a fonoaudiologia tenha avançado na personalização do atendimento e na adaptação das estratégias terapêuticas, ainda há um predomínio de intervenções centradas no diagnóstico e reabilitação, com número reduzido de estudos sobre ações de promoção da saúde e inclusão social.

## INTRODUÇÃO

A atuação e a importância do fonoaudiólogo na atenção à pessoa com Transtorno do Espectro do Autismo (TEA) é reconhecida como elemento central no desenvolvimento de habilidades comunicativas e sociais, que estão frequentemente comprometidas em indivíduos com essa condição^(1)^. O Transtorno do Espectro do Autismo é um transtorno do desenvolvimento caracterizado^(2,3)^ por déficits severos e persistentes nas habilidades de comunicação e interação social, além de comportamentos restritos e repetitivos, exigindo intervenções especializadas e individualizadas para abordar as especificidades de cada sujeito. Nesse contexto, os serviços de saúde desempenham papel crucial na organização e oferta de estratégias avaliativas e terapêuticas que assegurem um cuidado integral, contínuo e equitativo aos sujeitos^(4,5)^.

A fonoaudiologia, como disciplina essencial na abordagem interdisciplinar^(5)^ com crianças diagnosticadas com TEA, tem avançado no planejamento e na implementação de intervenções que incluem a necessária compreensão sobre o funcionamento integral do sujeito, sua família, cuidadores e educadores. Os profissionais fonoaudiólogos têm ampliado o seu acesso ao trabalho em diferentes espaços^(6)^ que desempenham papel multifacetado no sistema de saúde brasileiro, como em Centros de Saúde, Centros Especializados em Reabilitação (CER), Centros de Atenção Psicossocial (CAPS), Centros de Convivência e Cooperativa (CECCO), Clínicas Particulares, Clínicas-Escola, Organizações da Sociedade Civil (OSC), dentre outras possibilidades, podendo atuar a partir do conceito de níveis de complexidade a depender dos serviços oferecidos.

As intervenções realizadas em cada um dos espaços citados não apenas visam o desenvolvimento da comunicação funcional e das habilidades sociais dessas crianças, mas também contribuem para os processos de ampliação de condições para o acesso e a permanência nos espaços sociais, como a escola e o trabalho, e outras oportunidades de desenvolvimento e autonomia. No trabalho com crianças autistas recomenda-se^(4,5)^ que as práticas estejam relacionadas à personalização do cuidado, seja pela realização de avaliação para a identificação de habilidades comunicativas em defasagem, seja para o planejamento terapêutico individualizado sem, contudo, desconsiderar o contexto familiar e educacional do sujeito. Nesse decurso, a inclusão dos familiares^(4,5,7,8)^ no processo terapêutico deve ser realizada por meio de estratégias de orientações para a facilitação do convívio, da comunicação e da interação como elementos fundamentais para o sucesso do acompanhamento.

Nessa baila, a atuação do fonoaudiólogo nos diferentes níveis de complexidade de atenção à saúde contribui tanto com a mitigação das principais dificuldades de desenvolvimento de fala e de linguagem, quanto com a compreensão e construção de formas de práticas associadas aos processos sociais adjacentes ao desenvolvimento infantil^(3-5,8)^. Desse modo, a atuação do fonoaudiólogo nos diferentes níveis de complexidade de atenção à saúde é construída a partir das especificidades e demandas de cada sujeito, família e contexto, visando à promoção da saúde comunicativa e à integralidade^(9)^ do cuidado.

No cuidado à saúde, em especial nos primeiros anos^(5,8,9)^ de vida do sujeito, a atuação do fonoaudiólogo é estratégica para fortalecer a integralidade do cuidado, permitindo a detecção precoce de alterações, a implementação de medidas preventivas e a coordenação eficaz do cuidado nos demais níveis de atenção^(6)^, independentemente do espaço onde as ações são realizadas. Essa abordagem integrada contribui para a melhoria dos indicadores de saúde e para a promoção da qualidade de vida. Nesse âmbito, o fonoaudiólogo desempenha papel fundamental no cuidado, na promoção da saúde e na prevenção de dificuldades associadas ou convizinhas aos distúrbios da comunicação, incluindo o diagnóstico situacional das famílias e dos espaços sociais que permeiam a existência do seu paciente a fim de promover ações educativas, o atendimento individual ou coletivo (creches, escolas, espaços de convivência), e a realização de visitas domiciliares entre outras possíveis formas de atuação que podem ser construídas pelo profissional.

Embora os níveis de complexidade de atenção à saúde sejam amplamente discutidos no âmbito dos espaços e das práticas promovidas pelo Sistema Único de Saúde^(10)^, esse entendimento não é exclusivo dos serviços públicos. Permeia, nessa conceituação, o fundamento da atenção à saúde abrangente a todas as medidas voltadas ao bem-estar humano, englobando ações e serviços de promoção, prevenção, tratamento e reabilitação^(7)^ nos diferentes espaços e serviços, haja vista a complexa estrutura subjacente que compõe o sistema de saúde no Brasil; e a igualmente complexa dinâmica colaborativa entre instituições acadêmicas, OSCs e serviços públicos na promoção da saúde^(11)^.

Dada a crescente prevalência^(12-14)^ de TEA no mundo, e a complexidade de suas manifestações, torna-se imperativo compreender como a fonoaudiologia tem estruturado suas práticas terapêuticas nos diferentes contextos de atenção, quais são os espaços nos quais são recebidas as crianças autistas, suas famílias e cuidadores, e quais estratégias permeiam a práxis fonoaudiológica no cuidado nesse âmbito. Tendo em vista a ampliação da capacitação do fonoaudiólogo, é importante que os profissionais estejam preparados para depreender suas práticas afetadas e produzindo efeito nas políticas sociais de saúde desempenhando papel fundamental na forma como o autismo e outros transtornos são interpretados e abordados, refletindo as visões e perspectivas sociais sobre a criança autista e sobre o próprio autismo.

Nessa direção, este estudo apresenta uma revisão sistemática do tipo escopo^(15)^ que busca analisar quais práticas estão sendo relatadas por fonoaudiólogos, e caracterizar como essas práticas podem ser interpretadas enquanto modos de inserção e permanência de crianças autistas nos serviços de saúde. Especificamente, o objetivo desta revisão de escopo é identificar e sistematizar os modos como a prática fonoaudiológica para crianças autistas em serviços de saúde é relatada enquanto estratégia avaliativa e terapêutica no Brasil.

## MÉTODO

A pesquisa do tipo revisão de escopo foi realizada a partir das orientações PRISMA-ScR^(15,16)^ visando mitigar inconsistências na extração e análise dos dados. A revisão foi realizada entre março e julho de 2024. O protocolo de revisão foi registrado na plataforma *OSF - Open Science Framework* ^(17)^ com o código 9Z2VN.

A revisão se deu a partir da formulação de uma pergunta-guia que baseou a definição da estrutura P (população), C (conceito), C (contexto) de forma a construir objetivos claros e significativos, e critérios de elegibilidade. Pergunta: quais as características da prática fonoaudiológica com crianças autistas em serviços de saúde no Brasil? População: estudos publicados sobre crianças menores de 12 anos e com diagnóstico de transtorno do espectro autista. Conceito: estratégias de avaliação e terapia fonoaudiológica; Contexto: Brasil. A partir dessa definição foram instaurados critérios de inclusão e de exclusão de documentos elegíveis.

Como critérios de inclusão, obteve-se: documentos que relatam e/ou analisam tipos de práticas fonoaudiológicas, terapêuticas e avaliativas, realizadas no Brasil, com crianças e familiares em espaços de saúde; estudos que apresentam aprovação para a realização de pesquisa pelo Comitê de Ética em Pesquisa, quando se aplica; estudos que expressam o desenho da pesquisa de modo a ser possível classificar o desenho de estudo e identificar a população estudada, as estratégias de avaliação e terapia, e o contexto da realização do estudo. Foram incluídos estudos na íntegra e escritos nos idiomas português, inglês ou espanhol, sem limite de data de publicação.

Como critérios de exclusão, obteve-se: documentos que relatam práticas e estratégias fonoaudiológicas exclusivamente com adolescentes, adultos e/ou idosos; pesquisas que não foram realizadas por fonoaudiólogos; documentos que não relatam e/ou não analisam tipos de práticas fonoaudiológicas, terapêuticas e avaliativas. Foram excluídos estudos realizados em países distintos do Brasil e que não apresentam aprovação para a realização de pesquisa pelo Comitê de Ética em Pesquisa, quando se aplica; estudos que não expressam a estratégia da pesquisa de modo a ser possível classificar o desenho de estudo e identificar a população estudada, as estratégias de avaliação e terapia e o contexto da realização do estudo.

### Aspectos éticos

Este estudo compõe a fase um de pesquisa em desenvolvimento autorizada pelo Comitê de Ética em Pesquisa sob CAAE: 77227724.4.0000.5404.

### Estratégia de pesquisa

Uma vez realizadas as etapas anteriores, passou-se à definição dos descritores para a elaboração da sintaxe de busca dos estudos. Foram utilizados os vocabulários controlados DeCS (Descritores em Ciências da Saúde), MeSH (Medical Subject Headings), Títulos CINAHL (Cumulative Index to Nursing and Allied Health Literature), da EBSCOhost, e Emtree, para a pesquisa de descritores com características semânticas que acolhessem os objetivos da pesquisa. Foi realizada uma busca preliminar no PubMed para identificar artigos sobre o tema e refinar os termos de busca, incluindo variações terminológicas e termos livres relevantes. As bases de dados e portais utilizados para a revisão incluíram: PubMed; Scopus; Web of Science; Embase; BVS-Bireme; EBSCOhost; Proquest Central; Scielo; Google Acadêmico e BDTD. A estratégia de busca (Quadro 1) foi definida e realizada a partir da combinação de descritores e termos livres, adaptadas para cada base de dados.

**Quadro 1:** Estratégias de buscas nas bases de dados a partir da Matriz do Protocolo de revisão de escopo^(17)^

**Objetivo**	Identificar e sistematizar os modos como a prática fonoaudiológica com crianças autistas em serviços de saúde é relatada enquanto estratégia avaliativa e terapêutica no Brasil.				
**PCC**	**População (P)**	**Conceito ©**	**Contexto ©**		
**Limites**	**Crianças Autistas, de 2 a 12 anos**	**estratégias fonoaudiológicas de avaliação e terapia**	**Brasil**		
**Construção e Combinação**	**“Child”, “Child, Preschool”, “Autism Spectrum Disorder”, “Autistic Disorder”, “Asperger Syndrome”**	**Audiology, “Rehabilitative of Speech and Language Disorders”**	**“Speech, Language and Hearing Sciences”, “Speech Therapy”, Brazil OR Brasil Brazilian OR Brazilians**		
**Usado**	(Child OR “Child, Preschool”) AND (“Autism Spectrum Disorder” OR “Autistic Disorder” OR “Asperger Syndrome”) AND (“Speech, Language and Hearing Sciences” OR Audiology OR “Rehabilitation of Speech and Language Disorders” OR “Speech Therapy”) AND (Brazil OR Brasil or brazilian or Brazilians)				
**Fonte**	**Estratégia**			**Nº de Artigos**	**Data**
**BDTD**	(Todos os campos: Criança OR “Pré-Escolar” E Todos os campos:”Transtorno do Espectro Autista” OR “Transtorno Autístico” OR “Síndrome de Asperger” E Todos os campos:Fonoaudiologia OR Audiologia OR “Reabilitação dos Transtornos da Fala e da Linguagem” OR Fonoterapia E Todos os campos:BRASIL OR BRASILEIROS OR BRASILEIRAS OR BRASILEIRO OR BRASILEIRA)			16	04/07/2024
**BVS**	((child OR children) OR (“Child, Preschool” OR “Preschool Child” OR “Preschool Children”)) AND ((“Autism Spectrum Disorder” OR “Autism Spectrum Disorders” OR “Autistic Spectrum Disorder” OR “Autistic Spectrum Disorders”) OR (“Autistic Disorder” OR “Kanner’s Syndrome” OR “Kanner Syndrome” OR “Infantile Autism” OR Autismo OR “Early Infantile Autism”) OR (“Asperger Syndrome” OR “Asperger Disease” OR “Asperger Disorder” OR “Asperger Disorders” OR “Asperger’s Disorder” OR “Aspergers Disorder” OR “Asperger’s Syndrome” OR “Aspergers Syndrome”)) AND ((“Speech, Language and Hearing Sciences”) OR (audiology) OR (“Rehabilitation of Speech and Language Disorders” OR “Language and Speech Disorder Rehabilitation” OR “Speech and Language Disorder Rehabilitation”) OR (“Speech Therapy” OR “Speech Therapies”)) AND ((Brazil OR Brasil OR brazilian OR brazilians))			**14**	**04/07/2024**
**EBSCOHOST Academic Search Premier 5 MEDLINE 3** **MEDLINE Complete 3 CINAHL with Full Text 1 Computers & Applied Sciences Complete 1 ERIC 1 Library, Information Science & Technology Abstracts with Full Text 1**	(Child OR Children) OR (“Child, Preschool” OR “Preschool Child” OR “Preschool Children”) AND TI (“Autism Spectrum Disorder” OR “Autism Spectrum Disorders” OR “Autistic Spectrum Disorder” OR “Autistic Spectrum Disorders”) OR AB (“Autism Spectrum Disorder” OR “Autism Spectrum Disorders” OR “Autistic Spectrum Disorder” OR “Autistic Spectrum Disorders”) OR TI (“Autistic Disorder” OR “Kanner’s Syndrome” OR “Kanner Syndrome” OR “Infantile Autism” OR Autism OR “Early Infantile Autism”) OR AB (“Autistic Disorder” OR “Kanner’s Syndrome” OR “Kanner Syndrome” OR “Infantile Autism” OR Autism OR “Early Infantile Autism”) OR TI (“Asperger Syndrome” OR “Asperger Disease” OR “Asperger Disorder” OR “Asperger Disorders” OR “Asperger’s Disorder” OR “Aspergers Disorder” OR “Asperger’s Syndrome” OR “Aspergers Syndrome”) OR AB (“Asperger Syndrome” OR “Asperger Disease” OR “Asperger Disorder” OR “Asperger Disorders” OR “Asperger’s Disorder” OR “Aspergers Disorder” OR “Asperger’s Syndrome” OR “Aspergers Syndrome”) AND TI (“Speech, Language and Hearing Sciences”) OR AB (“Speech, Language and Hearing Sciences”) OR TI Audiology OR AB Audiology OR TI (“Rehabilitation of Speech and Language Disorders” OR “Language and Speech Disorder Rehabilitation” OR “Speech and Language Disorder Rehabilitation”) OR AB (“Rehabilitation of Speech and Language Disorders” OR “Language and Speech Disorder Rehabilitation” OR “Speech and Language Disorder Rehabilitation”) OR TI (“Speech Therapy” OR “Speech Therapies”) OR AB (“Speech Therapy” OR “Speech Therapies”) AND Brazil OR Brasil or brazilian or brazilians			09	04/07/2024
**EMBASE**	(‘child’ OR ‘children’ OR ‘child, preschool’ OR ‘preschool child’ OR ‘preschool children’) AND (‘autism spectrum disorder’:ti,ab,kw OR ‘autism spectrum disorders’:ti,ab,kw OR ‘autistic spectrum disorder’:ti,ab,kw OR ‘autistic spectrum disorders’:ti,ab,kw OR ‘autism’/exp OR ‘autistic disorder’:ti,ab,kw OR ‘kanner`s syndrome’:ti,ab,kw OR ‘kanner syndrome’:ti,ab,kw OR ‘infantile autism’:ti,ab,kw OR ‘autism’:ti,ab,kw OR ‘early infantile autism’:ti,ab,kw OR ‘asperger syndrome’/exp OR ‘asperger syndrome’:ti,ab,kw OR ‘asperger disease’:ti,ab,kw OR ‘asperger disorder’:ti,ab,kw OR ‘asperger disorders’:ti,ab,kw OR ‘asperger`s disorder’:ti,ab,kw OR ‘aspergers disorder’:ti,ab,kw OR ‘asperger`s syndrome’:ti,ab,kw OR ‘aspergers syndrome’:ti,ab,kw) AND (‘speech, language’ AND ‘hearing sciences’:ti,ab,kw OR ‘audiology’/exp OR ‘audiology’:ti,ab,kw OR (‘rehabilitation of speech’ AND ‘language disability’/exp) OR (((‘rehabilitation of speech’ AND ‘language disorders’:ti,ab,kw OR ‘language’) AND ‘speech disorder rehabilitation’:ti,ab,kw OR ‘speech’) AND ‘language disorder rehabilitation’:ti,ab,kw) OR ‘speech therapy’/exp OR ‘speech therapy’:ti,ab,kw OR ‘speech therapies’:ti,ab,kw) AND (‘brazil’ OR ‘brasil’ OR ‘brazilian’ OR ‘brazilians’)			35	04/07/2024
**Google Acadêmico**	(“Autismo” OR “Transtorno do Espectro Autista” OR TEA OR “Transtorno Autístico” OR “Síndrome de Asperger”) AND Fonoaudiologia AND BRASIL			47	04/07/2024
**PROQUEST CENTRAL**	((Child OR Children) OR (“Child, Preschool” OR “Preschool Child” OR “Preschool Children”)) AND (abstract(“Autism Spectrum Disorder” OR “Autism Spectrum Disorders” OR “Autistic Spectrum Disorder” OR “Autistic Spectrum Disorders”) OR title(“Autism Spectrum Disorder” OR “Autism Spectrum Disorders” OR “Autistic Spectrum Disorder” OR “Autistic Spectrum Disorders”) OR abstract(“Autistic Disorder” OR “Kanner’s Syndrome” OR “Kanner Syndrome” OR “Infantile Autism” OR Autism OR “Early Infantile Autism”) OR title(“Autistic Disorder” OR “Kanner’s Syndrome” OR “Kanner Syndrome” OR “Infantile Autism” OR Autism OR “Early Infantile Autism”) OR abstract(“Asperger Syndrome” OR “Asperger Disease” OR “Asperger Disorder” OR “Asperger Disorders” OR “Asperger’s Disorder” OR “Aspergers Disorder” OR “Asperger’s Syndrome” OR “Aspergers Syndrome”) OR title(“Asperger Syndrome” OR “Asperger Disease” OR “Asperger Disorder” OR “Asperger Disorders” OR “Asperger’s Disorder” OR “Aspergers Disorder” OR “Asperger’s Syndrome” OR “Aspergers Syndrome”)) AND (abstract(“Speech, Language and Hearing Sciences”) OR title(“Speech, Language and Hearing Sciences”) OR abstract(Audiology) OR title(Audiology) OR abstract(“Rehabilitation of Speech and Language Disorders” OR “Language and Speech Disorder Rehabilitation” OR “Speech and Language Disorder Rehabilitation”) OR title(“Rehabilitation of Speech and Language Disorders” OR “Language and Speech Disorder Rehabilitation” OR “Speech and Language Disorder Rehabilitation”) OR abstract(“Speech Therapy” OR “Speech Therapies”) OR title(“Speech Therapy” OR “Speech Therapies”)) AND (Brazil OR Brasil OR brazilian OR brazilians) https://www.proquest.com/search/2656553?accountid=8113			14	04/07/2024
**PUBMED**	(((((Child OR Children)) OR (“Child, Preschool” OR “Preschool Child” OR “Preschool Children”)) AND ((((((Autism Spectrum Disorder[MeSH Terms]) OR (“Autism Spectrum Disorder”[Title/Abstract] OR “Autism Spectrum Disorders”[Title/Abstract] OR “Autistic Spectrum Disorder”[Title/Abstract] OR “Autistic Spectrum Disorders”[Title/Abstract])) OR (Autistic Disorder[MeSH Terms])) OR (“Autistic Disorder”[Title/Abstract] OR “Kanner’s Syndrome”[Title/Abstract] OR “Kanner Syndrome”[Title/Abstract] OR “Infantile Autism”[Title/Abstract] OR Autism[Title/Abstract] OR “Early Infantile Autism”[Title/Abstract])) OR (Asperger Syndrome[MeSH Terms])) OR (“Asperger Syndrome”[Title/Abstract] OR “Asperger Disease”[Title/Abstract] OR “Asperger Disorder”[Title/Abstract] OR “Asperger Disorders”[Title/Abstract] OR “Asperger’s Disorder”[Title/Abstract] OR “Aspergers Disorder”[Title/Abstract] OR “Asperger’s Syndrome”[Title/Abstract] OR “Aspergers Syndrome”[Title/Abstract]))) AND (((((((“Speech, Language and Hearing Sciences”[Title/Abstract]) OR (Audiology[MeSH Terms])) OR (Audiology[Title/Abstract])) OR (Rehabilitation of Speech and Language Disorders[MeSH Terms])) OR (“Rehabilitation of Speech and Language Disorders”[Title/Abstract] OR “Language and Speech Disorder Rehabilitation”[Title/Abstract] OR “Speech and Language Disorder Rehabilitation”[Title/Abstract])) OR (Speech Therapy[MeSH Terms])) OR (“Speech Therapy”[Title/Abstract] OR “Speech Therapies”[Title/Abstract]))) AND (Brazil OR Brasil or brazilian or brazilians)			15	04/07/2024
**SCIELO**	(Criança OR Child OR Niño OR “Pré-Escolar” OR “Child, Preschool” OR Preescolar) AND (“Transtorno do Espectro Autista” OR “Autism Spectrum Disorder” OR “Trastorno del Espectro Autista” OR “Transtorno Autístico” OR “Autistic Disorder” OR “Transtorno Autístico” OR “Síndrome de Asperger” OR “Asperger Syndrome” OR “Síndrome de Asperger”) AND (Fonoaudiologia OR “Speech, Language and Hearing Sciences” OR Fonoaudiología OR Audiologia OR Audiology OR Audiología OR “Reabilitação dos Transtornos da Fala e da Linguagem” OR “Rehabilitation of Speech and Language Disorders” OR “Rehabilitación de los Trastornos del Habla y del Lenguaje” OR Fonoterapia OR “Speech Therapy” OR Logopedia) AND (Brasil OR Brazil OR brazilian or brazilians)			**03**	**04/07/2024**
**SCOPUS**	(((ALL (child OR children) OR ALL (“Child, Preschool” OR “Preschool Child” OR “Preschool Children”))) AND ((TITLE-ABS-KEY (“Autism Spectrum Disorder” OR “Autism Spectrum Disorders” OR “Autistic Spectrum Disorder” OR “Autistic Spectrum Disorders”) OR TITLE-ABS-KEY (“Autistic Disorder” OR “Kanner’s Syndrome” OR “Kanner Syndrome” OR “Infantile Autism” OR utismo OR “Early Infantile Autism”) OR TITLE-ABS-KEY (“Asperger Syndrome” OR “Asperger Disease” OR “Asperger Disorder” OR “Asperger Disorders” OR “Asperger’s Disorder” OR “Aspergers Disorder” OR “Asperger’s Syndrome” OR “Aspergers Syndrome”))) AND ((TITLE-ABS-KEY (“Speech, Language and Hearing Sciences”) OR TITLE-ABS-KEY (audiology) OR TITLE-ABS-KEY (“Rehabilitation of Speech and Language Disorders” OR “Language and Speech Disorder Rehabilitation” OR “Speech and Language Disorder Rehabilitation”) OR TITLE-ABS-KEY (“Speech Therapy” OR “Speech Therapies”)))) AND (ALL (brazil OR brasil OR brazilian OR brazilians))			**68**	**04/07/2024**
**WEB OF SCIENCE**	Child OR Children (Topic) or “Child, Preschool” OR “Preschool Child” OR “Preschool Children” (Topic) and Preprint Citation Index (Exclude – Database) AND”Autism Spectrum Disorder” OR “Autism Spectrum Disorders” OR “Autistic Spectrum Disorder” OR “Autistic Spectrum Disorders” (Topic) or “Autistic Disorder” OR “Kanner’s Syndrome” OR “Kanner Syndrome” OR “Infantile Autism” OR Autism OR “Early Infantile Autism” (Topic) or “Asperger Syndrome” OR “Asperger Disease” OR “Asperger Disorder” OR “Asperger Disorders” OR “Asperger’s Disorder” OR “Aspergers Disorder” OR “Asperger’s Syndrome” OR “Aspergers Syndrome” (Topic) and Preprint Citation Index (Exclude – Database) AND “Speech, Language and Hearing Sciences” (Topic) or Audiology (Topic) or “Rehabilitation of Speech and Language Disorders” OR “Language and Speech Disorder Rehabilitation” OR “Speech and Language Disorder Rehabilitation” (Topic) or “Speech Therapy” OR “Speech Therapies” (Topic) and Preprint Citation Index (Exclude – Database) AND Brazil OR Brasil or brazilian or brazilians (Topic) and Preprint Citation Index (Exclude – Database) https://www.webofscience.com/wos/alldb/summary/15f85a45-0b96-4f98-a3e9-85b2b8fa44a5-f9012fe9/relevance/1			**12**	**04/07/2024**
**TOTAL**				**233**	
**TOTAL DE REFERÊNCIAS EM DUPLICIDADE**	79 ARTIGOS EXCLUÍDOS POR DUPLICIDADES NO RAYYAN			**79+06=**	
	06 ARTIGOS EXCLUÍDOS POR DUPLICIDADES identificados na leitura (outro idioma)				
				**85**	
**TOTAL APÓS EXCLUSÃO DE DUPLICIDADE**				**148**	

### Seleção dos estudos

A etapa seguinte foi realizada utilizando o software Rayyan^(18)^ cujos arquivos dos artigos recuperados foram importados para a exclusão das duplicidades e depois triados pelo título e resumo. A seleção e a decisão de inclusão foram realizadas por dois pesquisadores, de forma independente e às cegas quanto à pertinência ou não da seleção e inclusão no estudo. Os conflitos, quando existiram, foram resolvidos a partir da análise de um terceiro revisor que realizou análise para definir a permanência ou a exclusão dos documentos. Foram incluídos todos os documentos disponíveis na íntegra e que cumprissem os critérios estabelecidos a partir do protocolo PRISMA^(16)^.

### Extração, mapeamento e apresentação dos resultados

Os dados foram obtidos conforme declarado em uma matriz^(17)^ de extração previamente elaborada a fim de observar autor, tipo e ano de publicação, objetivo e amostra populacional do estudo, conceito de autismo, tipo de intervenção, estratégias fonoaudiológicas realizadas e local de realização da pesquisa. Os dados foram analisados quantitativamente via análise descritiva utilizando medidas de tendência central e variabilidade. O teste de qui-quadrado, a 5% de significância, foi utilizado para a verificação da relação entre variáveis categóricas. Todas as análises e gráficos foram executados no software R^(19)^. A obtenção dos dados também passou por identificação qualitativa a partir da matriz^(17)^ de extração apresentada no protocolo da revisão.

## RESULTADOS

Como resultado das buscas, foram obtidos 233 arquivos. Após a seleção dos arquivos e remoção de duplicatas, 149 estudos foram lidos na íntegra, obtendo-se 92 elegíveis para análise. Foram excluídos outros 42 arquivos, o que resultou em 49 estudos incluídos na revisão para a extração de dados (Figura 1)^(16)^.

**Figura 1:** Diagrama de fluxo PRISMA – ScR 2020^18^ preenchido de acordo com os resultados da pesquisa

As publicações analisadas e as variáveis elencadas para análise estão apresentadas nos Quadro 2. A revisão de escopo revela que os estudos revisados têm como foco principal a avaliação da eficácia das intervenções fonoaudiológicas no desenvolvimento da comunicação e linguagem de crianças com Transtorno do Espectro Autista (TEA). A predominância desse tipo de investigação reflete a preocupação dos pesquisadores em validar abordagens terapêuticas específicas, bem como em compreender os impactos das estratégias utilizadas no engajamento comunicativo e na funcionalidade da linguagem das crianças autistas. Observa-se também um número significativo de estudos que buscam identificar o perfil comunicativo desses indivíduos, enfatizando tanto as características linguísticas e pragmáticas, quanto a percepção dos cuidadores sobre os progressos alcançados ao longo das intervenções.

**Quadro 2:** Dados do estudo preenchido a partir da matriz do protocolo de revisão de escopo para investigar as características de intervenção e estratégias fonoaudiológicas na atenção à criança autista em serviços de saúde

Referência	Tipo de Publicação	Ano de Publicação	Objetivo	População e Amostra	Conceito de Autismo	Tipo de Intervenção	Estratégias	Local de Intervenção	Conclusões
1. Balestro e Fernandes^(20)^	Artigo	2018	Analisar a percepção dos cuidadores de crianças com TEA quanto ao perfil funcional da comunicação de seus filhos por meio da aplicação do Perfil de Comunicação Funcional-Checklist (PFC-C).	CUIDADOR = 62 cuidadores de Crianças com TEA	DSM-IV	Terapêutica e Avaliativa	Protocolo e questionários	Clínica-escola	Foi confirmada a hipótese de que seria possível produzir mudanças positivas na perspectiva dos pais de crianças com TEA sobre a comunicação de seus filhos a partir do questionário aplicado.
2. Balestro e Fernandes^(21)^	Artigo	2012	Desenvolver e aplicar um questionário para levantamento das dificuldades percebidas por pais e/ou cuidadores de crianças com TEA na comunicação com seus filhos.	CUIDADOR = 40 pais e/ou cuidadores de crianças com TEA	Base-literatura	Avaliativa	Protocolo e questionários	Clínica-escola	A aplicação do questionário elaborado demonstrou utilidade na identificação de dificuldades comunicativas específicas da população alvo.
3. Balestro et al.^(22)^	Artigo	2009	Demonstrar a eficiência e eficácia de uma abordagem interacionista na terapia fonoaudiológica de linguagem de 3 sujeitos com TEA.	CRIANÇA = 3 Indivíduos com TEA de 6, 7 e 8 anos	Base-literatura	Terapêutica e Avaliativa	Contexto terapêutico Lúdico	Não informado	Os casos analisados demonstraram a eficácia da abordagem interacionista na terapêutica de linguagem dos sujeitos.
4. Barbosa et al.^(23)^	Artigo	2019	Identificar a percepção dos pais de crianças com TEA sobre o trabalho fonoaudiológico na equipe multiprofissional do CAPSij.	CUIDADOR = 9 pais de crianças com TEA de 2 a 10 anos	Base-literatura	Avaliativa	Grupo Focal	CAPSij	Na conversação dos pais foram claros os efeitos da escuta clínica oferecida pela equipe, em especial pelo fonoaudiólogo. A Fonoaudiologia foi considerada pertencente e fundamental ao campo da saúde mental no trabalho com crianças com autismo, o que reitera a escuta oferecida aos pais pela equipe.
5. Botura et al.^(24)^	Artigo	2021	Verificar a existência de alterações nas habilidades pragmáticas de crianças falantes de português brasileiro com TEA.	CRIANÇA	DSM-V	Não se aplica	Não se aplica	Não se aplica	Os achados foram inconclusivos.
6. Campelo et al.^(25)^	Artigo	2009	Analisar as habilidades comunicativas verbais e não verbais de crianças autistas através de um modelo de análise que toma como base os atos comunicativos, identificando-se os meios e funções comunicativas.	CRIANÇA = 6 Indivíduos com TEA do sexo masculino entre 4 e 10 anos	DSM-IV	Avaliativa	Contexto terapêutico Lúdico	Clínica-escola	Apareceram mais atos comunicativos por meio gestual em detrimentos dos meios vocais e verbais. Houve grande variedade, com predominância das funções não focalizadas, protesto, exploratória e reativa.
7. Cardoso e Montenegro^(26)^	Artigo	2009	Determinar o perfil comunicativo funcional de dois grupos de sujeitos com TEA e comparar o perfil comunicativo funcional de dois grupos.	CRIANÇA = 8 crianças e adolescentes com TEA entre 3 e 17 anos	DSM-IV	Terapêutica e Avaliativa	Contexto terapêutico Lúdico	Clínica-escola	Ainda que uma diferença tenha sido identificada entre o grupo que passou pela troca de terapeuta e o que não passou, essa diferença não é estatisticamente significativa, o que pode ser atribuído à heterogeneidade dos indivíduos e ao pequeno tamanho da amostra.
8. Castro e Loureiro^(27)^	Artigo	2020	Revisão integrativa da literatura sobre musicoterapia e fonoaudiologia no aspecto da interação social e linguagem de crianças com TEA.	CRIANÇA	DSM-IV	Não se aplica	Não se aplica	Não se aplica	Foram encontrados 14 estudos que abordaram as interfaces entre fonoaudiologia e musicoterapia na intervenção de crianças com TEA à respoeuto da comunicação e interação social. Os resultados obtidos foram positivos, sugerindo que a música favorece a expressão da linguagem oral, melhorando o prognóstico do paciente.
9. Costa^(28)^	Dissertação [mestrado]	2021	Investigar o desempenho da linguagem de crianças pré-escolares com diagnóstico de TEA e comparar com crianças com desenvolvimento típico de linguagem por meio da aplicação da versão brasileira do *Preschool Language Assessment Instrument* (PLAI-2).	CRIANÇA = 15 Indivíduos com TEA entre 3 anos e 5 anos e 11 meses	DSM-V	Avaliativa	Protocolo e questionários	Clínica-escola	O grupo amostral apresentou desempenho inferior nas habilidades avaliadas quando comparado ao grupo comparativo, que apresentou melhor desempenho.
10. Costa et al.^(29)^	Artigo	2013	Traduzir para o português do Brasil e adaptar culturalmente o teste *Children's Checklist 2* (CCC-2), bem como avaliar sua consistência interna.	CUIDADOR	Base-literatura	Não se aplica	Não se aplica	Não se aplica	O estudo apresentou o processo de tradução e adaptação transcultural do CCC-2. Porém, para que o teste possa ser amplamente utilizado em ambientes clínicos e de pesquisa, ainda há necessidade de validação da versão brasileira do instrumento.
11. Cruz e Gomes^(30)^	Trabalho de Conclusão de Curso (graduação)	2020	Identificar na literatura as principais intervenções fonoaudiológicas em crianças com TEA.	CRIANÇA	DSM-V	Não se aplica	Não se aplica	Não se aplica	Foi constatado que, por meio de intervenções diretas e indiretas, há maior evolução nos marcos de desenvolvimento de tais crianças. Assim, valida-se que a formação parental é essencial no processo terapêutico da criança com TEA, de modo a favorecer o envolvimento familiar.
12. Cruz e Tamanaha^(31)^	Artigo	2021	Analisar interações envolvendo a participação de sujeitos autistas não verbais ancorado em uma abordagem multimodal da interação humana.	CRIANÇA = 2 Indivíduos com TEA de 7 anos não verbais	DSM-V	Terapêutica e Avaliativa	Abordagem multimodal PECS	Não informado	A atenção do analista para o que acontece corporalmente é crucial para captar a maneira como crianças autistas orientam-se para a complexa e dinâmica ecologia interacional.
13. Netrval^(32)^	Tese [doutorado]	2014	Elaborar um modelo de indicadores de qualidade para o gerenciamento do serviço de Fonoaudiologia oferecido nas instituições que atendem a indivíduos com TEA.	CRIANÇA	DSM-V	Avaliativa	Protocolo e questionários	Multicêntrico	O questionário aplicado permitiu que se conhecesse mais sobre um grupo de indivíduos com TEA assistidos no município de São Paulo e o perfil dos locais que oferecem essa assistência. Os dados levantados demonstram que o planejamento terapêutico singular ainda é pouco utilizado e que os profissionais pouco discutem sobre o gerenciamento deste acompanhamento.
14. Ferreira et al.^(33)^	Artigo	2023	Descrever programas de intervenção precoce no TEA, analisando e correlacionando a equipe multi/interprofissional, o papel da Fonoaudiologia e a participação da família no cuidado.	CRIANÇA E CUIDADOR	DSM-V	Não se aplica	Não se aplica	Não se aplica	O atendimento especializado e precoce a crianças com TEA e seus familiares não tem estabelecido um padrão metodológico único, existindo uma variedade de abordagens terapêuticas em diferentes países, apesar da predominância do método Early Start Denver.
15. Santos et al.^(34)^	Artigo	2020	Analisar o impacto da implementação do Picture Exchange Communication System (PECS) na compreensão de instruções de crianças com TEA.	CRIANÇA = 20 Indivíduos com TEA entre 6 e 12 anos de idade	DSM-V	Terapêutica e Avaliativa	Abordagem multimodal PECS	Clínica-escola	Foi confirmada a hipótese de que o uso do PECS tenha efeito positivo na compreensão de instruções, uma vez que proporcionou às crianças maior engajamento comunicativo e social.
16. Deliberato et al.^(35)^	Artigo	2021	Descrever a mediação do adulto e as habilidades comunicativas de crianças com TEA em situações lúdicas, por meio da narração de histórias.	CRIANÇA = 2 Indivíduos com TEA do sexo masculino com 2 e 3 anos	Base-literatura	Terapêutica e Avaliativa	Contexto terapêutico Lúdico	Clínica-escola	A pesquisa mostrou que o uso da narração de histórias pode ser um caminho importante para as crianças com TEA no que diz respeito à interação e à comunicação.
17. Donadio et al.^(36)^	Artigo	2023	Medir como o *Prompts for Restructuring Oral Muscular Phonetic Targets* (PROMPT) melhora a fala de crianças com TEA.	CRIANÇA = 1 Indivíduo com TEA do sexo masculino de 6 anos	DSM-V	Terapêutica e Avaliativa	Método Prompt	Clínica-escola	Foi verificado que o participante apresentou melhorias no controle da fala, mandibular, labio-facial e lingual.
18. Evangelista^(37)^	Trabalho de Conclusão de Curso (graduação)	2018	Determinar a importância da fonoaudiologia no processo de cuidado a pacientes com TEA na Rede de Saúde Mental.	CRIANÇA	Base-literatura	Não se aplica	Não se aplica	Não se aplica	Observou-se que existe uma grande lacuna de publicações que abordem a temática da saúde mental sob a ótica da Fonoaudiologia, além do fato que a inserção da Fonoaudiologia se mostra incipiente e fragmentada.
19. Fernandes et al.^(38)^	Artigo	2010	Verificar a interferência do uso de computadores e programas específicos na terapia fonoaudiológica de crianças autistas.	CRIANÇA = 23 Indivíduos com TEA entre 3 e 12 anos	Base-literatura	Terapêutica e Avaliativa	Jogos de Informática	Clínica-escola	Os sujeitos apresentaram reações diferentes à proposta de utilização dos recursos de informática durante a terapia fonoaudiológica.
20. Fernandes et al.^(39)^	Artigo	2008	Verificar a existência de diferenças observáveis a partir das características do perfil funcional da comunicação e do desempenho sócio-cognitivo de crianças e adolescentes com TEA.	CRIANÇA = 46 Indivíduos com TEA entre 7 e 9 anos	Base-literatura	Terapêutica e Avaliativa	Oficina de Linguagem	Clínica-escola	Os resultados não indicaram diferenças estatisticamente significativas a partir das características do perfil funcional da comunicação e do desempenho sócio-cognitivo, embora observáveis.
21. Fernandes et al.^(40)^	Artigo	2010	Verificar a interferência de orientações a mães de crianças autistas sobre os processos de comunicação e linguagem, perfil comunicativo e desempenho sócio-cognitivo; e verificar a interferência dessas orientações sobre a forma com que as mães observam o desempenho de seus filhos.	DÍADE FAMILIAR-CRIANÇA = 26 díades mãe-criança com TEA	Base-literatura	Orientação parental	Grupos de orientação	Clínica-escola	Há hipótese de que orientações sistematizadas e específicas realizadas por curtos períodos de tempo e com possibilidades de retorno podem contribuir para o ambiente comunicativo da criança autista e para o entendimento familiar.
22. Ferreira et al.^(41)^	Artigo	2021	Analisar a repercussão da implementação do PECS no índice de sobrecarga de mães de crianças com TEA.	DÍADE FAMILIAR-CRIANÇA = 20 díades mãe-criança com TEA	DSM-V	Terapêutica e Avaliativa	Abordagem multimodal PECS	Clínica-escola	Houve tendência de redução dos índices de sobrecarga materna após a implementação do PECS.
23. Freitas et al.^(42)^	Artigo	2021	Descrever as habilidades de comunicação de crianças com TEA considerando a perspectiva clínica e familiar.	CUIDADOR = 10 pais e 04 terapeutas	DSM-V	Avaliativa	Protocolo e questionários	Clínica-escola	Verificou-se que ambos, pais e terapeutas, referiram déficits tanto nas habilidades comunicativas verbais quanto nas não verbais das crianças com TEA.
24. Gomes et al.^(43)^	Artigo	2004	Verificar se o comportamento clínico da hipersensibilidade auditiva relatado em entrevistas com pais, cuidadores e terapeutas de crianças autistas corresponde a achados audiológicos, e determinar se outros tipos de estímulo também levam à hipersensibilidade. auditiva.	CRIANÇA E ADOLESCENTE = 46 crianças, adolescentes e jovens adultos com TEA entre 05 e 20 anos	DSM-IV	Avaliativa	Protocolo e questionários	Clínica-escola	Manifestações comportamentais aos sons não estão associadas a hipersensibilidade das vias auditivas, mas às dificuldades de processamento no córtex cerebral, envolvendo sistemas geralmente prejudicados no TEA, como o sistema límbico.
25. Kamita et al.^(44)^	Artigo	2020	Identificar e analisar quais são os achados característicos dos Potenciais Evocados Auditivos Corticais (PEAC) em crianças e/ou adolescentes com TEA em comparação com o desenvolvimento típico.	CRIANÇA E ADOLESCENTE	DSM-V	Não se aplica	Não se aplica	Não se aplica	A população com TEA pode apresentar respostas diversas para os componentes dos PEAC em comparação ao desenvolvimento típico.
26. Kwee et al.^(45)^	Artigo	2009	Aplicar o protocolo de avaliação transdisciplinar baseado do Programa *Treatment and Education of Autistic and related Communication handicapped Children* – TEACCH a fim de observar o desenvolvimento e realizar os devidos ajustes nos programas de seis dos seus alunos.	CRIANÇA = 6 Indivíduos com TEA de 7 a 12 anos	Base-literatura	Terapêutica e Avaliativa	Comportamental	Clínica-escola	Observou-se que todos os alunos do programa obtiveram evolução positiva em todas as áreas e que apesar da complexidade das suas estruturas, os ganhos e as manutenções dos comportamentos adquiridos foram conquistados e estabilizados.
27. Martins e Fernandes^(46)^	Artigo	2013	Avaliar mudanças no Perfil Funcional da Comunicação (PFC) e no Desempenho Sócio-Cognitivo (DSC) de crianças com Distúrbios do Espectro do Autismo (DEA) a partir de dois períodos curtos de intervenção.	CRIANÇA = 21 Indivíduos com TEA entre 2 e 12 anos	DSM-V	Terapêutica e Avaliativa	Intervenção de curta duração	Clínica-escola	Com a estrutura de intervenção em 12 semanas, foi possível observar poucas mudanças no PFC e no DSC, de forma que o estudo sugere novas pesquisas com a duração mais longa.
28. Matas et al.^(47)^	Artigo	2009	Descrever os resultados audiológicos e eletrofisiológicos de indivíduos com distúrbios psiquiátricos, procurando distúrbios auditivos periféricos e/ou centrais.	CRIANÇA E ADOLESCENTE = 40 Indivíduos com TEA entre 8 e 19 anos	DSM-IV	Avaliativa	Protocolo e questionários	Não informado	Uma alta ocorrência de alterações auditivas foi verificada em crianças com desordens psiquiátricas, embora, em algumas análises, não tenha diso observada diferença estatisticamente significativa quando o grupo amostral foi comparado com o grupo controle.
29. Mayerle et al.^(48)^	Artigo	2021	Analisar a *Mismatch Negativity* (MMN) (resposta cerebral a alterações auditivas devido à memória) em crianças e adolescentes com TEA e compará-los com um grupo controle.	CUIDADOR = 68 cuidadores de crianças e adolescentes com TEA.	DSM-V	Avaliativa	Protocolo e questionários	Clínica-escola	Crianças e adolescentes com TEA possuem maiores valores de latência e amplitude no MMN do que os indivíduos do grupo controle.
30. Menezes e Perissinoto^(49)^	Artigo	2008	Avaliar a habilidade de atenção compartilhada em sujeitos com TEA em diferentes contextos e com diferentes interlocutores.	CRIANÇA = 20 crianças com TEA	DSM-IV	Terapêutica e Avaliativa	Contexto terapêutico Lúdico	Clínica-escola	A avaliação da atenção compartilhada em contexto de brincadeira foi eficaz e a intervenção do adulto refletiu no aumento destes comportamentos nas situações semi-dirigidas e de imitação.
31. Miilher e Fernandes^(50)^	Artigo	2009	Analisar a evolução dos aspectos funcionais e gramaticais em três momentos distintos: avaliação inicial, após seis meses de terapia e após doze meses de terapia.	CRIANÇA = 10 crianças com TEA do sexo masculino entre 2 anos e 7 meses e 11 anos e 2 meses	DSM-IV	Terapêutica e Avaliativa	Contexto terapêutico Lúdico	Clínica-escola	Há relação entre o desempenho gramatical e pragmático.
32. Miilher e Fernandes^(51)^	Artigo	2012	Comparar o perfil pragmático das iniciativas de comunicação e o perfil bidimensional envolvendo os aspectos de iniciativa e responsividade.	CRIANÇA = 30 gravações de terapia fonoaudiológica de 10 pacientes crianças com TEA.	DSM-IV	Terapêutica e Avaliativa	Contexto terapêutico Lúdico	Clínica-escola	Os resultados evidenciaram a necessidade de considerar o perfil bidimensional de comunicação e ressaltaram a necessidade de qualificar as respostas a fim de discriminar as habilidades comunicativas da criança.
33. Misquiatti et al.^(52)^	Artigo	2014	Analisar o desempenho sociocognitivo de crianças e adolescentes com TEA em dois ambientes de terapia de linguagem, que se diferenciam quanto à estruturação do aspecto físico	CRIANÇA E ADOLESCENTE = 10 crianças e adolescentes com TEA entre 4 e 13 anos	Base-literatura	Terapêutica e Avaliativa	Contexto terapêutico Lúdico	Clínica-escola	A criação de ambientes físicos preestabelecidos ou materiais específicos não são e não devem ser considerados imprescindíveis para a terapia de linguagem.
34. Misquiatti et al.^(53)^	Artigo	2015	Avaliar a sobrecarga de familiares cuidadores de crianças com TEA, segundo a percepção dos próprios cuidadores.	CUIDADOR = 10 familiares de crianças com TEA, e 10 familiares de crianças com transtornos de linguagem	DSM-IV	Avaliativa	Protocolo e questionários	Não informado	Os resultados observados mostraram que não houve diferença quanto à média do índice de sobrecarga do familiar cuidador de crianças com TEA e de crianças com transtornos de linguagem.
35. Misquiatti e Fernandes^(54)^	Artigo	2011	Analisar o perfil funcional comunicativo de crianças e adolescentes com TEA em dois ambientes diferentes de terapia de linguagem, com as diferenças concentradas no aspecto físico do ambiente.	CRIANÇA = 10 crianças e adolescentes com TEA entre 4 e 13 anos	Base-literatura	Terapêutica e Avaliativa	Enriquecimento ambiental	Clínica-escola	O ambiente físico estudado não teve influência significativa no perfil funcional comunicativo dos sujeitos autistas, ainda que houvesse indivíduos com tendência de apresentar melhor performance em um ambiente em relação a outro.
36. Moraes^(55)^	Dissertação [mestrado]	2019	Verificar a efetividade de um programa de estimulação à distância aplicado no LIFDEA-FMUSP.	CRIANÇA E ADOLESCENTE =30 crianças e adolescentes com TEA entre 6 e 16 anos	DSM-V	Terapêutica e Avaliativa	Telefonoaudiologia	Clínica-escola	Constatou-se que a quantidade de estratégias propostas para um mesmo objetivo de intervenção terapêutica pode variar de forma a se perceber a grande variedade de possibilidades para trabalhar as características do distúrbio do espectro do autismo.
37. Neubauer e Fernandes^(56)^	Artigo	2013	Verificar a utilização de um checklist, em substituição ao protocolo completo, para analisar o Perfil Funcional da Comunicação (PFC).	CRIANÇA = filmagens de terapia fonoaudiológica de 50 crianças com TEA entre 3 e 12 anos	DSM-IV	Avaliativa	Contexto terapêutico Lúdico	Clínica-escola	O checklist pode ser usado como instrumento de acompanhamento de processos terapêuticos de crianças com TEA, mas não substitui o instrumento completo.
38. Navarro^(57)^	Artigo	2018	Reinterpretar a prática fonoaudiológica no contexto da Equoterapia com base na Neurolinguística Discursiva (ND).	CRIANÇA = 1 criança TEA, sexo masculino	DSM-V	Terapêutica e Avaliativa	Equoterapia	Não informado	A reinterpretação possibilitada pela ND sobre o acompanhamento fonoaudiológico no contexto da Equoterapia expande e explica os acontecimentos da cena equoterapêutica como um todo.
39. Pereira et al.^(58)^	Artigo	2019	Verificar os efeitos da intervenção fonoaudiológica com Comunicação Aumentativa e Alternativa (CAA) nos atos comunicativos em crianças com TEA.	CRIANÇA = 3 crianças com TEA do sexo masculino entre 2 e 4 anos	DSM-V	Terapêutica e Avaliativa	Abordagem multimodal PECS	Clínica-escola	O uso da CAA na clínica fonoaudiológica mostra-se promissor e eficaz no que se refere à promoção do desenvolvimento das habilidades comunicacionais do indivíduo com TEA.
40. Roncati et al.^(59)^	Artigo	2019	Replicar os procedimentos de Coon and Miguel (histórias de reforço com uma topografia de alerta específica) com crianças com TEA, que são comumente expostas ao tato e avisos ecóicos ao aprender o comportamento verbal.	CRIANÇA = 3 crianças com TEA do sexo masculino de 3, 6 e 7 anos	Base-literatura	Terapêutica e Avaliativa	Método Prompt	Não informado	Para os profissionais que planejam aulas para ensinar comportamento intraverbal a crianças com TEA, informações sobre o histórico de um paciente com um tipo de prompt específico pode ser essencial.
41. Rosa^(60)^	Dissertação [mestrado]	2022	Investigar o desempenho da narrativa oral de história em crianças e adolescentes com TEA sem deficiência intelectual (DI).	CRIANÇA = 27 indivíduos do sexo masculino com TEA sem deficiência intelectual	Base-literatura	Avaliativa	Protocolo e questionários	Clínica-escola	O grupo amostral obteve desempenho semelhante a indivíduos com desenvolvimento típico de linguagem na compreensão oral de histórias e superior na produção narrativa oral de histórias.
42. Santos et al.^(61)^	Artigo	2022	Investigar a contribuição da Telefonoaudiologia no desenvolvimento das habilidades de comunicação de crianças com TEA durante a pandemia da COVID-19.	CRIANÇA = 8 crianças com TEA entre 2 e 8 anos	DSM-V	Avaliativa	Telefonoaudi-ologia	Não informado	Os resultados demonstraram que a teleconsulta fonoaudiológica para crianças com TEA durante o isolamento social, decorrente da pandemia pela COVID-19, mostrou-se uma opção viável e que produziu avanços na comunicação das crianças.
43. Segeren e Fernandes^(62)^	Artigo	2019	Pesquisar a casuística proporcionada por um serviço de saúde de referência.	CRIANÇA = Prontuários dos pacientes que realizaram terapia fonoaudiológica no LIF-DEA.	DSM-V	Não se aplica	Não se aplica	Clínica-escola	Mostrou a necessidade de registros sistemáticos do trabalho realizado, preferencialmente com bancos de dados informatizados. Foi observada uma limitação na caracterização dos dados e redução da perda desses dados após a digitalização.
44. Silva et al.^(63)^	Artigo	2007	Descrever o processo de intervenção fonoaudiológica de uma criança com diagnóstico de autismo.	CRIANÇA = Uma criança com TEA do sexo masculino de 2 anos	DSM-IV	Terapêutica e Avaliativa	Contexto terapêutico Lúdico	Clínica-escola	As estratégias terapêuticas aqui apresentadas mostraram-se eficazes para a criança em questão, uma vez que esta apresentou evolução frente ao processo terapêutico fonoaudiológico.
45. Sun et al.^(64)^	Artigo	2017	Identificar métodos efetivos para estimular a linguagem e comunicação de crianças com TEA, o que é fundamental para usar efetivamente os recursos disponíveis para o suporte dessas crianças.	CRIANÇA = 20 crianças com TEA entre 5 e 12 anos	Base-literatura	Terapêutica e Avaliativa	Contexto terapêutico Lúdico	Clínica-escola	A intervenção com inclusão de atividades de estimulação das funções executivas para crianças com TEA requer mais investigações.
46. Tamanaha e Perissinoto^(65)^	Artigo	2011	Analisar e comparar a extensão e a velocidade do processo evolutivo de crianças com TEA assistidas em intervenção terapêutica fonoaudiológica direta e indireta em detrimento à indireta.	CRIANÇA = 11 crianças de 4 a 10 anos do sexo masculino com TEA e Síndrome de Asperger	DSM-IV	Terapêutica e Avaliativa	Contexto terapêutico Lúdico	Clínica-escola	A tendência de melhor desempenho das crianças assistidas em ambas as intervenções mostrou que a associação de ações diretas e indiretas é fundamental no processo terapêutico fonoaudiológico de crianças com TEA.
47. Tamanaha et al.^(66)^	Artigo	2008	Avaliar o processo evolutivo da criança autista em contexto de intervenção direta e indireta a partir das respostas das mães ao *Autism Behavior Checklist.*	CUIDADOR = 11 mães de crianças com TEA ou Síndrome de Asperger	DSM-IV	Terapêutica e Avaliativa	Contexto terapêutico Lúdico	Clínica-escola	Tanto no grupo terapêutico direto (GT) quanto no indireto (GO) as mães observaram mudanças de comportamentos. A tendência de escores melhores do GT deveu-se, provavelmente, à eficácia da intervenção direta e não à falta de atenção dos pais do GO .
48. Tamanaha et al.^(67)^	Artigo	2015	Avaliar a eficácia da intervenção terapêutica fonoaudiológica para crianças com Distúrbios do Espectro do Autismo.	CRIANÇA = 11 Crianças TEA com retardo mental leve a moderado	DSM-V	Terapêutica e Avaliativa	Protocolo e questionários	Clínica-escola	Concluiu-se que a associação de intervenções diretas e indiretas é fundamental para o melhor desempenho dessas crianças.
49. Santos^(68)^	Tese [doutorado]	2017	Verificar a eficácia de instrumentos (PFC-C, FCP-Rr - TP, FCP-Rr - OF, PFC, ToPS) para a investigação das habilidades pragmáticas de crianças com TEA.	CRIANÇA = 30 crianças com TEA	DSM-V	Terapêutica e Avaliativa	Protocolo e questionários	Clínica-escola	Os protocolos FCP-Rr e PFC-C foram sensíveis e consistentes na verificação das habilidades pragmáticas de indivíduos com autismo a partir das respostas das terapeutas.

A relação entre os objetivos dos estudos e suas conclusões reforça a eficácia de diferentes estratégias terapêuticas. Intervenções como o *Picture Exchange Communication System* - (PECS) e a musicoterapia foram relatadas como ferramentas que favorecem o engajamento comunicativo, proporcionando avanços no desenvolvimento da linguagem e na interação social. Além disso, os estudos indicam que abordagens interativas e contextos terapêuticos lúdicos desempenham um papel central na melhora das habilidades comunicativas das crianças. Observou-se também, apesar de relatada em menor número de publicações, a importância da orientação parental e da escuta clínica, evidenciada por pesquisas que analisaram o impacto das intervenções no cotidiano das famílias, destacando a necessidade de envolvimento ativo dos cuidadores no processo terapêutico.

Quanto à “população e amostra”, os estudos envolveram predominantemente crianças diagnosticadas com TEA com idade entre 02 e 12 anos de idade. Contudo, há associações de pesquisa com adolescentes e jovens adultos em alguns estudos (Quadro 2). Em alguns casos, foram analisados também os cuidadores das crianças, especialmente quando o foco da pesquisa era observar o impacto da orientação familiar sobre o processo terapêutico. Além disso, a comparação entre crianças e adolescentes, observadas em alguns estudos, permitiu uma visão mais ampla sobre a progressão das habilidades linguísticas ao longo do desenvolvimento da linguagem e da comunicação.

Quanto ao detalhamento desses dados (Tabela 1), obteve-se que das publicações revisadas 85% são do tipo artigo^(20-27,29,31,33-36,38-54,56-59,61-67)^; 6,1% dissertações^(28,55,60)^; 4,1% teses^(32,68)^; e 4,1% são trabalhos de conclusão de curso^(30,37)^. A maior parte dos trabalhos, 65,3%, foi publicada entre os anos 2013 e 2024^(20,23,24,27,29,31,33-59,61,62,64,67,68)^ (Tabela 1), coincidindo com o período de publicação da atualização do Manual Diagnóstico Estatístico de Transtornos Mentais - DSM-V, e 34,7% entre os anos 2004 e 2012^(20,21,25,26,38-40,43,45,47,49-51,54,63,65,66)^.

**Tabela 1:** Distribuição de frequências dos “tipos de publicação”, “conceito de autismo”, “tipo de intervenção” e “local de intervenção” e “estratégias de intervenção”

	Frequência absoluta	Frequência percentual
**Tipo de publicação**		
Artigos	42	85,7%
Dissertação [Mestrado]	3	6,1%
Tese [Doutorado]	2	4,1%
Trabalho de Conclusão de Curso [Graduação]	2	4,1%
**Conceito de autismo**		
DSM-V	20	40,8%
Base_literatura	15	30,6%
DSM-IV	14	28,6%
**Tipo de intervenção**		
Terapêutica e Avaliativa	27	67,5%
Avaliativa	12	30,0%
Orientação parental	1	2,5%
**Local de Intervenção**		
Clínica-escola	33	78,6%
Não-informado	7	16,7%
CAPSij	1	2,4%
Multicêntrico	1	2,4%
**Estratégias de intervenção**		
Contexto terapêutico Lúdico	12	30,0%
Protocolo e questionários	12	30,0%
Abordagem multimodal PECS	4	10,0%
Método Prompt	2	5,0%
Telefonoaudiologia	2	5,0%
Comportamental	1	2,5%
Enriquecimento ambiental	1	2,5%
Equoterapia	1	2,5%
Grupo Focal	1	2,5%
Grupos de orientação	1	2,5%
Intervenção de curta duração	1	2,5%
Jogos de Informática	1	2,5%
Oficina de Linguagem	1	2,5%

O resultado da extração dos dados apresentou relação entre os estudos publicados por fonoaudiólogos no Brasil e os períodos de publicação e atualização do DSM. O DSM-V é citado em 40,8% das publicações^(24,28,30,34,41,42,44,46,48,55,57,58,61,62,67,68)^, o DSM-IV em 28,6%^(20,25-27,43,47,49-51,53,56,63,65,66)^, e 30,6% os citam indiretamente utilizando outras publicações (base literatura)^(20-23,29,35,37-40)^ para descrever os sintomas do autismo. O teste de independência de qui-quadrado aponta que a utilização do conceito de autismo depende da data de publicação do estudo (p-value < 0,001) (Tabela 2). Entre 2004 e 2012 o conceito de autismo na formulação das pesquisas adveio majoritariamente do DSM-IV, enquanto, de 2013 a 2024 passou a ser o DSM-V. Conceitos baseados na literatura existente (base literatura) foi igualmente usada nesses dois períodos.

**Tabela 2:** Tabela de contingência (cruzamentos) entre: “tipo de intervenção” x “local de intervenção”, “Ano de publicação” x “conceito de autismo” e “tipos de intervenção” x “estratégias de intervenção”, ressaltando o valor-p do teste de independência

Tipo de intervenção	CAPSij	Clínica-escola	Não-informado	Valor-p
Avaliativa	1	8	3	
Orientação parental	0	1	0	0,7142
Terapêutica e Avaliativa	0	23	4	
Conceito de autismo	2004-2012	2013-2024		
Base-literatura	7	8		
DSM-IV	10	4		<0,001
DSM-V	0	20		
Estratégias de intervenção	Avaliativa	Orientação parental	Terapêutica e Avaliativa	
Abordagem multimodal PECS	0	0	4	
Comportamental	0	0	1	
Contexto terapêutico Lúdico	1	0	11	
Enriquecimento ambiental	0	0	1	
Equoterapia	0	0	1	
Grupo Focal	1	0	0	
Grupos de orientação	0	1	0	<0,01
Intervenção de curta duração	0	0	1	
Jogos de Informática	0	0	1	
Método Prompt	0	0	2	
Oficina de Linguagem	0	0	1	
Protocolo e questionários	8	0	3	
Telefonoaudiologia	1	0	1	

Dos estudos revisados, o “tipo de intervenção” predominante foi a abordagem “terapêutica e avaliativa”, evidenciando o foco no tratamento e na mensuração dos avanços das crianças ─ considerando que na categoria “terapêutica e avaliativa” foram incluídas publicações que objetivam fazer levantamento e/ou análise avaliativa e associam práticas terapêuticas; na categoria “avaliativa” foram incluídas publicações que visam apenas procedimentos avaliativos; na categorias “orientação parental” foram incluídas publicações que relatam práticas exclusivas com pais e/ou cuidadores, excluindo-se práticas de avaliação –, obteve-se que a maioria das publicações, 67,5%^(20,22,26,31,34-36,38-40,45,46,49-52,54,55,57-59,63-68)^, apresentou o “tipo de intervenção” associado a práticas terapêuticas e avaliativas, 30%^(2,23,25,28,42,43,47,48,53,56,60,61)^ relata prática apenas avaliativa, e apenas 2,5%^(69)^ apresentou prática desenvolvida com orientação parental (Tabela 1).

Na categoria “local de intervenção”, 78,6% das publicações^(20,21,25,26,28,34-36,38-43,45,46,48-52,54,55,57,58,60,62-68)^ relatam práticas desenvolvidas em clínica-escola de universidades públicas e privadas; 2,4% foi desenvolvido em CAPSij^(23)^; 2,4% relata ação multicêntrica^(32)^; e 16,7% discriminam o local da prática como serviço de saúde^(24,27,29-31,33,35,44,47,53,57,59,61)^, mas sem especificação. Ou seja, a grande maioria das publicações relata práticas associadas ao exercício de avaliação ou reabilitação, majoritariamente realizado em clínica-escola.

Em relação à comparação dos dados coletados (Tabela 2), o teste de independência de qui-quadrado aponta que o local de intervenção não depende do tipo de intervenção realizada (p-value = 0,7142). Isto é, independentemente do local de intervenção, o tipo de intervenção mais frequente é “Terapêutica e Avaliativa”. Por outro lado, a clínica-escola é o local mais frequente, independentemente do tipo de intervenção.

Os dados extraídos sobre as “estratégias de intervenção” empregadas nas intervenções variaram amplamente, incluindo protocolos padronizados, questionários, grupos focais, oficinas de linguagem e métodos baseados no lúdico. Há também o relato de abordagens interativas, como o uso de histórias narrativas e jogos de informática. Estudos que investigaram a influência do ambiente terapêutico apontaram que a estrutura física e a organização dos espaços podem afetar a responsividade e o desempenho das crianças nas interações comunicativas. A “estratégia terapêutica” mais relatada nas publicações foi (Tabela 1) “contexto terapêutico lúdico”^(22,25,26,35,49-52,63-66)^, com frequência percentual de 30%, igualmente ao uso de “protocolo e questionários”^(20,21,28,32,42,43,47,48,53,60,67,68)^. A “abordagem multimodal com uso de PECS”^(31,34,41,58)^ aparece em 10%, seguida de 5% com “método Prompt”^(36,59)^, 5% com “telefonoaudiologia”^(55,61)^, 2,5% “abordagem comportamental”^(45)^, 2,5% “enriquecimento ambiental”^(54)^, 2,5% “equoterapia”^(57)^, 2,5% “grupo focal”^(23)^, 2,5% “intervenção de curta duração”^(46)^, 2,5% “jogos de informática”^(38)^, e 2,5% “oficina de linguagem”^(39)^.

Quando realizado o cruzamento entre “tipos de intervenção” x “estratégias” (Tabela 2), ressalta-se o *p-value* <0,01 informando que a estratégia terapêutica depende do tipo de intervenção priorizada. Quando a intervenção é apenas “avaliativa”, a estratégia preferida é a utilização de “protocolo e questionários”. Porém, quando é “terapêutica e avaliativa”, a estratégia é majoritariamente no “contexto terapêutico lúdico”. Estratégias terapêuticas como o contexto terapêutico lúdico e o uso de protocolos e questionários são amplamente relatadas. Observa-se que a abordagem lúdica é preferida em intervenções terapêuticas, enquanto protocolos padronizados predominam em estudos focados exclusivamente na avaliação.

## DISCUSSÃO

A predominância de estudos voltados à avaliação da eficácia das intervenções fonoaudiológicas reflete a preocupação dos pesquisadores em validar abordagens terapêuticas e compreender os impactos das estratégias utilizadas no engajamento comunicativo das crianças autistas. A concentração das práticas em clínicas-escola sugere uma tendência dos estudos revisados em focar no tratamento e avaliação, em detrimento de ações de promoção à saúde em contextos comunitários.

A transição do DSM-IV para o DSM-V como referencial predominante nas pesquisas sobre autismo reflete a filiação paradigmática dos pesquisadores na compreensão do transtorno dentro dessa comunidade científica. Embora essa transição tenha sido impulsionada, em grande parte, por perspectivas biologicistas, tradicionais e focadas na identificação de déficits^(70)^, é importante considerar que essa abordagem não esgota as possibilidades de entendimento do autismo. Diferentes correntes teóricas e metodológicas contribuem para a construção do conhecimento na área, e a predominância de um modelo não implica na exclusão de outros.

O DSM^(71,72)^ foi criado para auxiliar profissionais da saúde mental no diagnóstico de transtornos mentais. Sua primeira edição, o DSM-I, foi publicada em 1952 *pela American Medico-Psychological Association*, posteriormente chamada de *American Psychiatric Association* (APA). Desde então, o manual passou por quatro revisões: DSM-II, DSM-III, DSM-IV e DSM-V. Ao longo do tempo, a compreensão sobre o autismo passou por transformações que acompanharam avanços científicos, mudanças nas atitudes sociais e aprimoramentos nos métodos de diagnóstico^(70,73)^. Inicialmente, o autismo era confundido com outras condições psiquiátricas e, posteriormente, foi reconhecido como um transtorno de espectro específico. A história da conceituação do autismo mostra a dificuldade de se obter consenso^(74,75)^, mas, o DSM, dispositivo criado pela APA, encontra ampla concordância entre profissionais clínicos e pesquisadores, no Brasil. A identificação da condição autista e a descrição dos sintomas é baseada, no país, nas definições que constam nesse dispositivo^(72)^.

Embora o DSM seja uma ferramenta essencial para a padronização do diagnóstico e comunicação entre profissionais, ele também recebe críticas^(8,21,76-78)^ por sua rigidez e tendência a negligenciar a subjetividade dos indivíduos autistas. Além disso, argumentam sobre os rígidos critérios que podem negligenciar a diversidade dos indivíduos autistas. Os autores relatam e se apoiam no fato de o autismo ser uma variação da neurodiversidade humana, e não um transtorno a ser padronizado ou corrigido. Nesse sentido, a especialização excessiva e a ampliação dos critérios diagnósticos poderiam contribuir para uma “epidemia de autismo”. De outro lado, outros pesquisadores defendem que a manutenção do Manual Diagnóstico e Estatístico de Transtornos Mentais (DSM) é essencial para o diagnóstico e terapia do autismo, pois fornece um sistema de classificação padronizado que facilita a comunicação entre pesquisadores, médicos e formuladores de políticas.

O DSM-V trouxe avanços no refinamento diagnóstico ao agrupar os transtornos do espectro autista sob um único diagnóstico e enfatizar critérios clínicos que incluem déficits na comunicação e interação social, bem como padrões restritos e repetitivos de comportamento. No entanto, essa abordagem^(79-81)^, centrada nos critérios médicos e neurobiológicos, pode, em alguns contextos, limitar o entendimento sobre a complexidade social e fenomenológica, e das experiências vividas por indivíduos autistas, uma perspectiva enfatizada por abordagens sociais e da neurodiversidade. Além disso, a persistência do uso de conceitos fundamentados na literatura existente demonstra a coexistência de diferentes abordagens interpretativas. Pesquisas que utilizam referenciais teóricos interdisciplinares, como os estudos sociocognitivos, pragmáticos e da educação inclusiva, apontam para a necessidade de ampliar o escopo de investigação, incorporando perspectivas que vão além da classificação e do diagnóstico^(78)^. Essas abordagens ressaltam, por exemplo, o papel do contexto social, da comunicação alternativa e aumentativa, e da interação com os ambientes nos processos de desenvolvimento e aprendizado das crianças autistas.

O uso dos critérios diagnósticos delimitados pela APA pode ter direcionado o modo como os estudos revisados construíram suas práticas com crianças com autismo. As descrições relatam práticas executadas em ambientes mais relacionados ao cuidado reabilitador do que em ambientes voltados ao cuidado de promoção à saúde. Ampla maioria dos estudos revelam práticas executadas em clínicas-escola. A predominância de intervenções “Terapêuticas e Avaliativas” em ambientes como clínicas-escola reflete a tendência em concentrar suas ações no tratamento e avaliação de crianças com Transtorno do Espectro Autista (TEA). Ou seja, a concentração dessas práticas em ambientes clínicos e acadêmicos limita a abrangência das ações fonoaudiológicas, especialmente no que tange à promoção da saúde e à prevenção em contextos comunitários.

Nessa mesma direção, o cruzamento entre “tipos de intervenção” e “estratégias” demonstrou que a escolha das estratégias terapêuticas depende do tipo de intervenção adotada (p-value < 0,01). Intervenções exclusivamente avaliativas tendem a utilizar protocolos estruturados, enquanto as terapêuticas combinadas preferem contextos interativos e lúdicos. Se de um lado, a avaliação^(79,80)^ clínica fonoaudiológica é importante para estabelecer o diagnóstico funcional para a construção de um plano de intervenção que atenda às necessidades específicas de cada paciente, por outro lado a promoção da saúde, que também cabe e deve ser realizada em ambientes clínicos, visa ampliar as possibilidades de cuidado frente às diferentes faces da vida individual e social^(82)^. No contexto do TEA, isso implicaria em ações que favorecessem a inclusão social, a conscientização pública e o apoio às famílias, aspectos que podem ser subestimados quando o foco recai predominantemente em espaços, tipos de intervenções e estratégias terapêuticas tradicionais.

A análise das “conclusões” das pesquisas revisadas sugere que algumas investigações não conseguiram estabelecer diferenças estatisticamente significativas entre os grupos avaliados, possivelmente devido à heterogeneidade das amostras e ao pequeno número de participantes. Além disso, há ênfase em estudos conduzidos em ambientes clínico-escolares, o que limita a compreensão dos efeitos das intervenções em contextos comunitários de saúde e familiares. Em muitos casos, as pesquisas sugerem que intervenções mais prolongadas e abrangentes seriam necessárias para captar mudanças sutis e sustentáveis na comunicação das crianças com TEA.

Assim, a revisão das conclusões aponta para a importância de ampliar o foco relativamente ao espaço e “tipo de intervenção” das pesquisas na área da fonoaudiologia, incluindo a diversidade de contextos sociais e comunicacionais de crianças com TEA.

## CONCLUSÃO

Esta pesquisa do tipo revisão de escopo permitiu identificar e sistematizar as práticas fonoaudiológicas voltadas para crianças autistas em serviços de saúde no Brasil, evidenciando a predominância de estudos que descrevem abordagens terapêuticas e avaliativas desenvolvidas majoritariamente em clínicas-escola. A análise dos estudos revelou que, embora a fonoaudiologia tenha avançado na personalização do atendimento e na adaptação das estratégias terapêuticas em espaços de saúde pela diversificação das abordagens terapêuticas, e pela preferência por estratégias lúdicas e contextuais, ainda predominam intervenções centradas no diagnóstico e reabilitação.

A diversidade de estratégias identificadas aponta para a possibilidade de estender as práticas de modo a considerar não apenas as dificuldades dos sujeitos assistidos, mas também a necessidade de incluir práticas que considerem a relação do sujeito com seu contexto familiar, educacional e social

Contudo, é importante ressaltar que o estudo realizado se limita à estratégia de busca realizada neste protocolo de revisão. Diante disso, recomenda-se a ampliação das investigações sobre práticas comunitárias e multiprofissionais, bem como a realização de novos estudos que explorem diferentes níveis de atenção à saúde, permitindo melhor compreensão sobre o cuidado fonoaudiológico no TEA. O fortalecimento da articulação entre saúde, educação e assistência social também deve ser incentivado, promovendo um modelo de atenção mais integral, que contemple tanto a avaliação e o tratamento, quanto a inclusão e a promoção da qualidade de vida social das crianças autistas e de suas famílias.

A predominância na frequência percentual de estudos realizados em clínicas-escola aponta para uma sub-representação de práticas desenvolvidas em espaços como Unidades Básicas de Saúde (UBS), Centros de Convivência e Centros de Atenção Psicossocial Infantil (CAPSij), limitando a visão sobre a diversidade das intervenções aplicadas em serviços públicos. Essa lacuna evidencia a necessidade de ampliar a diversidade metodológica e os cenários de pesquisa, incorporando investigações que analisem a efetividade das estratégias terapêuticas em diferentes realidades e ao longo do desenvolvimento das crianças.

O estudo sugere que o relato de práticas fonoaudiológicas em ambientes comunitários de saúde é escassa, instaurando uma lacuna na atenção à criança autista. A integração de abordagens interdisciplinares, como práticas colaborativas entre fonoaudiólogos, educadores e psicólogos, pode ampliar a efetividade das intervenções e proporcionar suporte abrangente para as crianças e suas famílias.
